# Intimate partner violence in 46 low-income and middle-income countries: an appraisal of the most vulnerable groups of women using national health surveys

**DOI:** 10.1136/bmjgh-2019-002208

**Published:** 2020-01-26

**Authors:** Carolina V N Coll, Fernanda Ewerling, Claudia García-Moreno, Franciele Hellwig, Aluisio J D Barros

**Affiliations:** 1 International Center for Equity in Health, Universidade Federal de Pelotas, Pelotas, RS, Brazil; 2 Reproductive Health and Research, WHO, Geneve, GE, Switzerland

**Keywords:** epidemiology, public health, community-based survey

## Abstract

**Introduction:**

Intimate partner violence (IPV) against women is a critical public health issue that transcends social and economic boundaries and considered to be a major obstacle to the progress towards the 2030 women, children and adolescents’ health goals in low-income and middle-income countries (LMICs). Standardised IPV measures have been increasingly incorporated into Demographic and Health Surveys carried out in LMICs. Routine reporting and disaggregated analyses at country level are essential to identify populational subgroups that are particularly vulnerable to IPV exposure.

**Methods:**

We examined data from 46 countries with surveys carried out between 2010 and 2017 to assess the prevalence and inequalities in recent psychological, physical and sexual IPV among ever-partnered women aged 15–49 years. Inequalities were assessed by disaggregating the data according to household wealth, women’s age, women’s empowerment level, polygyny status of the relationship and area of residence.

**Results:**

National levels of reported IPV varied widely across countries—from less than 5% in Armenia and Comoros to more than 40% in Afghanistan. Huge inequalities within countries were also observed. Generally, richer and more empowered women reported less IPV, as well as those whose partners had no cowives. Different patterns across countries were observed according to women’s age and area of residence but in most cases younger women and those living in rural areas tend to be more exposed to IPV.

**Conclusion:**

The present study advances the current knowledge by providing a global panorama of the prevalence of different forms of IPV across LMICs, helping the identification of the most vulnerable groups of women and for future monitoring of leaving no one behind towards achieving the elimination of all forms of violence among women and girls.

**Key questions**

**What is already known?**

- Intimate partner violence (IPV) is among the most common forms of violence against women globally and can lead to profound, long-lasting and wide-ranging health implications for survivors.
- The Sustainable Development Goals call for gender equality and the empowerment of women and girls, bringing the need for more rigorous monitoring of IPV levels and the identification of those most in need of interventions.

**What are the new findings?**

- We found huge inequalities in IPV levels across low-income and middle-income countries (LMICs). Poorer, younger and less empowered women are particularly vulnerable to IPV exposure in most countries, as well as women whose partners had other cowives and those living in rural areas.
- Inequalities tend to be higher for physical and/or sexual IPV in comparison with psychological IPV.

**What do the new findings imply?**

- The present study provides a global panorama of IPV levels across LMICs using comparable data that can serve as a benchmark for future monitoring on leaving no one behind towards achieving the elimination of all forms of violence among women and girls.
- The analytical approaches presented here, with the disaggregation of data according to relevant equity dimensions, provide a deeper understanding of patterns of inequalities and thus help policy makers to set priority groups for their policies and programmes.
- Continuous data collection and monitoring of IPV levels will be essential to track progress and assess the impact of prevention and response efforts that have been implemented in LMICs.

## Introduction

Violence against women, including intimate partner violence (IPV), is a major obstacle to the fulfilment of women’s human rights and to the achievement of the Sustainable Development Goals (SDGs). This is particular the case in low-income and middle-income countries (LMICs) where the prevalence tends to be higher.^1^ IPV contemplates any behaviour within an intimate relationship that causes physical, psychological or sexual harm to those in the relationship.^2^ According to WHO estimates, nearly one-third of women aged 15 years and older around the world have experienced physical or sexual violence at the hands of an intimate partner in their lifetimes, with even higher proportions found in Africa and South East Asia.^3^

IPV exposure significantly impacts the health and well-being of women by increasing the risk of adverse outcomes and risk behaviours such as depressive symptoms, suicidal thoughts and attempts, alcohol and drug use, unwanted pregnancies, abortions and sexual transmitted infections.^3–5^ Women exposed to IPV are less likely to receive adequate antenatal and skilled delivery care than women who have not experienced abuse.^6^ There is also growing evidence that IPV and child maltreatment can co-occur within households and produce intergenerational effects.^7–9^ Children of mothers experiencing IPV are under a higher risk of under-five mortality, poor growth and development, as well as to an increased risk of perpetrating or experiencing IPV against women later in life.^10–13^

Given its widespread, profound and long-lasting consequences for survivors and families, the international community has increasingly recognised the urgent need to improve global policy action to tackle violence against women.^14 15^ In this context, the inclusion of a specific target on eliminating of all forms of violence against women and girls within the 2030 Agenda for Sustainable Development—under the goal of achieving gender equality and empowering all women and girls—was central to increasing commitment by governments. Despite the growing international attention, however, there is still limited investment in IPV research and coordination in measuring progress towards the 2030 SDGs in most LMICs.^16^

Routine reporting and disaggregated analyses at country level are essential to identify populational subgroups that are particularly vulnerable to IPV exposure, helping the implementation of targeted evidence-based prevention and response programming. For the present analysis, we estimated recent IPV levels across LMICs and inequalities according to household wealth, women’s age, women’s empowerment level, polygyny status of the partnership and area of residence.

## Methods

We used data from Demographic and Health Surveys (DHS) conducted in LMICs between 2010 and 2017 that included the ‘domestic violence module’ and assessed IPV using a structured questionnaire, following the WHO guidelines for the conduct of IPV research. These guidelines emphasise individual informed consent and the importance of ensuring confidentiality and privacy to improve the quality of the data and guarantee the safety of the respondent. Therefore, women only answer the questions on the violence module if the ideal conditions are met.^17^ All women aged 15–49 years who were usual residents of the selected household or who slept in the households the night before the survey were eligible for individual interviews with the full woman’s questionnaire. Given the sensitivity of the questions, a subsample of women was selected for the violence module (one eligible woman per household). For the present analysis, IPV estimates were generated at the country level by reanalysing the original survey data. Sample weights were used to adjust for within-household selection and non-response, ensuring that the domestic violence subsample was nationally representative. The ethical responsibility for the DHS lies with the institutions that conducted the surveys in each country; we, therefore, did not require ethics approval for this study.

The current prevalence of recent IPV was defined as the proportion of ever-partnered women aged 15–49 years who reported having experienced at least one act of IPV by a current or former intimate partner in the past 12 months, independently of the frequency. Questions asked to the women were: ‘*Did your (last) husband ever do any of the following things to you?*’ and ‘*How often* did this happen during the last 12 months: often, only sometimes, or not at all?’. The acts presented to participants to assess the occurrence of each type of IPV are summarised in box 1. Estimates were calculated separately for psychological, physical and sexual IPV. A combined indicator of having experienced physical or sexual IPV, or both, was also calculated for comparability with previous publications on the topic.^1^

Box 1 **List of situations presented to the women to assess IPV occurrence in the past 12 months. The alternatives are presented to the women with the question ‘did your partner…’**

**Physical violence**

- Push you, shake you or throw something at you.
- Slap you.
- Twist your arm or pull your hair.
- Punch you with fist or hit with something that could hurt you.
- Kick you, drag you, or beat you up.
- Try to choke you or burn you on purpose.
- Threaten or attack you with a gun, knife or other weapon.

**Sexual violence**

- Physically force you to have sexual intercourse with him when you did not want to.
- Physically forced you to perform any other sexual act when you did not want.
- Force you with threats or in any other way to perform sexual acts you did not want to.

**Psychological violence**

- Say or do something to humiliate you in front of others.
- Threaten to hurt or harm you or someone you cared about.
- Insult you or make to feel bad about herself.

Note: although part of the IPV scope, controlling behaviours such as isolating a person from family and friends and restricting access to financial resources were not considered in the present analysis.

### Equity stratifiers

To identify the most vulnerable groups of women in each country, national-level estimates were disaggregated by some factors that have been consistently found to predict IPV risk and distribution.^18 19^ Availability across datasets, comparability between countries and potential for policy impact across settings were also considered in the selection process. For the present analyses, we examined IPV inequalities according to household wealth in quintiles, women’s age, women’s empowerment level (attitude to violence), polygyny status of the relationship and area of residence.

#### Wealth

The classification of households according to socioeconomic position is based on a wealth index based on the ownership of household appliances (such as televisions and refrigerators) and other assets (as cattle and vehicles) and on characteristics of the building (materials used for walls, floor and roof and presence of electricity, water supply and sanitary facilities) derived through principal components analysis.^20 21^ Because relevant assets and their importance may vary in urban and rural households, separate indices are derived for each area. They are then combined into a single score using a scaling procedure. The resulting index is a comparable measure of wealth for urban and rural areas.^22^ Households are then classified into quintiles of the resulting score, where Q1 includes the 20% poorest households and Q5 the 20% richest households of each country.

Absolute wealth inequalities were estimated using the Slope Index of Inequality (SII). The SII represents the difference, in percentage points, between the fitted values of the IPV prevalence for the top and bottom of the wealth distribution. The index is expressed on a scale of −100 to +100, where zero represents the equitable distribution of the attribute on the wealth scale, a positive value means the outcome is concentrated towards the rich and a negative value means the outcome is concentrated towards the poor. More details on its calculation can be found elsewhere.^23^

#### Women’s empowerment (attitude to violence)

Women’s empowerment level in relation to their attitude to violence was assessed based on the Survey-based Women’s emPowERment (SWPER) Index.^24 25^ The SWPER global is an individual-level indicator based on 14 questions present in most DHS that allow the assessment of three empowerment domains indicative of assets and agency (attitude to violence, social independence and decision making) and considered a suitable common measure of women’s empowerment in the context LMICs.^25^ As for the other domains, all 14 questions are taken into account during the index validation procedure, but the attitude to violence domain is dominated by five questions regarding the woman’s opinion on whether it is justified that a husband beats his wife in specific situations (woman goes out without telling the husband; neglects the children; argues with husband; refuses to have sex; and burns the food).^25^ The attitude to violence domain of the SWPER is closely related to the concept of intrinsic agency, as a proxy for the woman’s incorporation of gender norms related to wife beating. In the presented analysis, we aimed to explore how violence acceptance (assessed by the women’s opinion on whether wife beating was justified) is related to IPV exposure. Following the SWPER methodology,^25^ women were categorised into low, medium and high empowerment level—with high empowerment meaning more attitude against violence—based on their SWPER scores.

### Other factors

Survey data were also stratified by woman’s age (15–19 years, 20–34 years and 35+ years), polygyny status of the partnership based on the woman’s report on the number of their partner co-wives (no cowives; 1+ cowives) and place of residence (urban/rural area) according to the classification of the sampled clusters by the national government at the time of the survey. Analyses were stratified by polygyny and women’s empowerment whenever the survey provided information on these stratifiers.

The analyses were performed with Stata V.15.1). All the analyses considered the surveys’ sample design.

### Patient and public involvement

Patients were not involved.

## Results

Data from a total of 372 149 ever-partnered women aged 15–49 years from 46 LMICs were assessed. table 1 presents the prevalence of the different forms of IPV in each country, organised by world region (UNICEF). Recent IPV estimates varied widely between countries. The prevalence of psychological IPV ranged from 6.2% in Comoros to 34.4% in Afghanistan while the proportion of women reporting physical and/or sexual IPV ranged from 3.5% in Armenia to 46.0% in Afghanistan. Large inequalities were also observed between countries of the same region. In South Asia, as an example, physical and/or sexual IPV varied from 5.5% in the Maldives to 46.0% in Afghanistan. Countries that stood out for presenting higher levels of both psychological and physical and/or sexual IPV—close to or above 30%—were Cameroon (32.1% and 31.4%) and Congo DR (29.4% and 36.7%) in West and Central Africa; Mozambique (29.6% and 27.7%), Tanzania (28.1% and 29.5%) and Uganda (29.3% and 29.6%) in Eastern and South Africa; Afghanistan (34.4% and 46.0%) in South Asia; and Colombia (30% and 33.3%) in Latin America and Caribbean.

**Table 1:** Prevalence of recent intimate partner violence (IPV) among ever-partnered women aged 15–49 years by country, grouped by world region

Country	ISO	Year	IPV in the past 12 months (%)				Sample size
			Physical	Sexual	Psychological	Physical and/or sexual	
West and Central Africa (12 countries)							
Burkina Faso	BFA	2010	8.9	1.1	7.2	9.2	10 009
Cameroon	CMR	2011	28.0	11.2	32.1	31.4	4006
Chad	TCD	2014	15.5	6.8	16.3	17.4	3814
Congo DR	COD	2013	30.3	19.8	29.4	36.7	5691
Cote d’Ivoire	CIV	2011	21.3	5.7	16.1	23.0	5018
Gabon	GAB	2012	28.3	11.8	26.6	31.2	4147
Gambia	GMB	2013	6.9	1.1	8.5	7.3	3542
Mali	MLI	2012	20.7	12.1	26.2	26.6	3120
Nigeria	NGA	2013	9.3	3.7	15.3	10.9	22 305
Senegal	SEN	2017	8.9	5.9	9.4	12.2	2660
Sierra Leone	SLE	2013	27.2	5.1	20.8	28.6	4315
Togo	TGO	2013	10.7	4.8	24.1	12.7	5376
Eastern and Southern Africa (14 countries)							
Angola	AGO	2015	24.2	6.7	24.0	25.8	7669
Burundi	BDI	2016	17.9	18.4	16.5	27.8	7366
Comoros	COM	2012	4.2	1.3	6.2	4.8	2529
Ethiopia	ETH	2016	16.9	8.3	20.2	19.7	4720
Kenya	KEN	2014	22.6	9.8	23.8	25.4	4519
Malawi	MWI	2015	16.2	15.4	23.0	24.1	5406
Mozambique	MOZ	2011	25.9	6.9	29.6	27.7	5824
Namibia	NAM	2013	18.7	6.6	21.0	20.2	1449
Rwanda	RWA	2014	17.6	8.3	18.5	20.6	1908
South Africa	ZAF	2016	8.7	3.5	10.9	10.4	2354
Tanzania	TZA	2015	27.0	10.4	28.1	29.5	7597
Uganda	UGA	2016	22.3	16.4	29.3	29.6	7536
Zambia	ZMB	2013	21.3	13.0	17.8	26.5	9416
Zimbabwe	ZWE	2015	15.2	9.3	23.5	19.8	5800
Middle East and North Africa (two countries)							
Egypt	EGY	2014	13.5	2.7	13.1	14.0	6693
Jordan	JOR	2017	12.7	3.3	16.1	13.8	6852
Europe and Central Asia (three countries)							
Armenia	ARM	2015	3.5	0.3	6.4	3.5	3540
Kyrgyzstan	KGZ	2012	16.9	2.8	10.4	17.1	4832
Tajikistan	TJK	2017	18.7	1.4	13.3	19.0	5313
South Asia (five countries)							
Afghanistan	AFG	2015	45.8	6.1	34.4	46.0	21 324
India	IND	2015	22.5	5.2	11.4	24.1	66 013
Maldives	MDV	2016	5.4	0.7	7.6	5.5	3388
Nepal	NPL	2016	10.0	4.0	7.7	11.2	3826
Pakistan	PAK	2017	13.6	3.6	20.6	14.5	3303
East Asia and Pacific (four countries)							
Cambodia	KHM	2014	9.3	3.9	17.3	10.9	3499
Myanmar	MMR	2015	10.2	2.2	10.2	11.0	3425
Philippines	PHL	2017	4.3	2.2	12.9	5.4	13 215
Timor Leste	TLS	2016	33.1	4.8	8.9	34.6	3694
Latin America and Caribbean (six countries)							
Colombia	COL	2015	32.3	7.6	30.0	33.3	24 890
Dominican Republic	DOM	2013	14.7	4.2	25.6	15.6	5803
Guatemala	GTM	2014	7.9	2.6	14.4	8.5	6512
Haiti	HTI	2016	10.0	7.0	17.8	13.8	4322
Honduras	HND	2011	10.0	3.2	20.6	10.9	12 494
Peru	PER	2016	10.2	2.5	10.5	10.8	21 115

In the South Africa DHS Survey, the violence module was administered to women aged 18 years or older. IPV estimates are, therefore, for women aged 18–49 years.

DHS, Demographic and Health Surveys; ISO, International Organization for Standardization.

On the other extreme, levels below 10% for both psychological and physical and/or sexual IPV were observed in Burkina-Faso (7.2% and 9.2%) and Gambia (8.5% and 7.3%) in West and Central Africa; Comoros (6.2% and 4.8%) in Eastern and South Africa, Armenia (6.4% and 3.5%) in Europe and Central Asia and Maldives (7.6% and 5.5%) in South Asia.

Despite the substantial overlap between both IPV indicators (Pearson’s correlation=0.74), in some countries IPV levels varied substantially depending on the nature of the violence reported. In Timor-Leste, for example, 8.9% of the women reported having experienced psychological IPV while 34.6% reported having experienced physical and/or sexual IPV. However, the prevalence of psychological IPV was 25.6% in Dominican Republic while physical and/or sexual IPV was 15.6%.

IPV prevalence by wealth quintiles can be checked at online supplementary data, table S1, which also presents the absolute inequality measured by the SII. Overall, greater wealth inequalities were observed for physical and/or sexual IPV than for psychological IPV. Most countries presented negative values of absolute inequality (higher IPV prevalence among the poorer women) for both IPV indicators. Togo (SII=−20.8), Cambodia (SII=−18.7), Uganda (SII=−16.1), Pakistan (SII=−16.2), Dominican Republic (SII=−14.8), Jordan (SII=−7.9) and Tajikistan (SII=−8.6) presented the highest level of inequalities for psychological IPV in their respective regions. For physical and/or sexual IPV, countries presenting higher wealth inequalities in their regions are India (SII=−26.4), Gabon (SII=−24.9), Uganda (SII=−23.1), Timor-Leste (SII=−19.9), Dominican Republic (SII=−15.5), Tajikistan (SII=−15.6) and Egypt (SII=−6.6). The opposite pattern, with IPV prevalence concentrated towards the rich, was only observed for a few African countries. In West and Central Africa, Nigeria, Cote d’Ivoire and Sierra Leone presented a positive SII for both psychological and physical and/or sexual IPV while in Burkina-Faso a positive SII was only observed for physical and/or sexual IPV. This was also the case in Angola from Eastern and Southern Africa.

10.1136/bmjgh-2019-002208.supp1 Supplementary data

**Figure 1:**
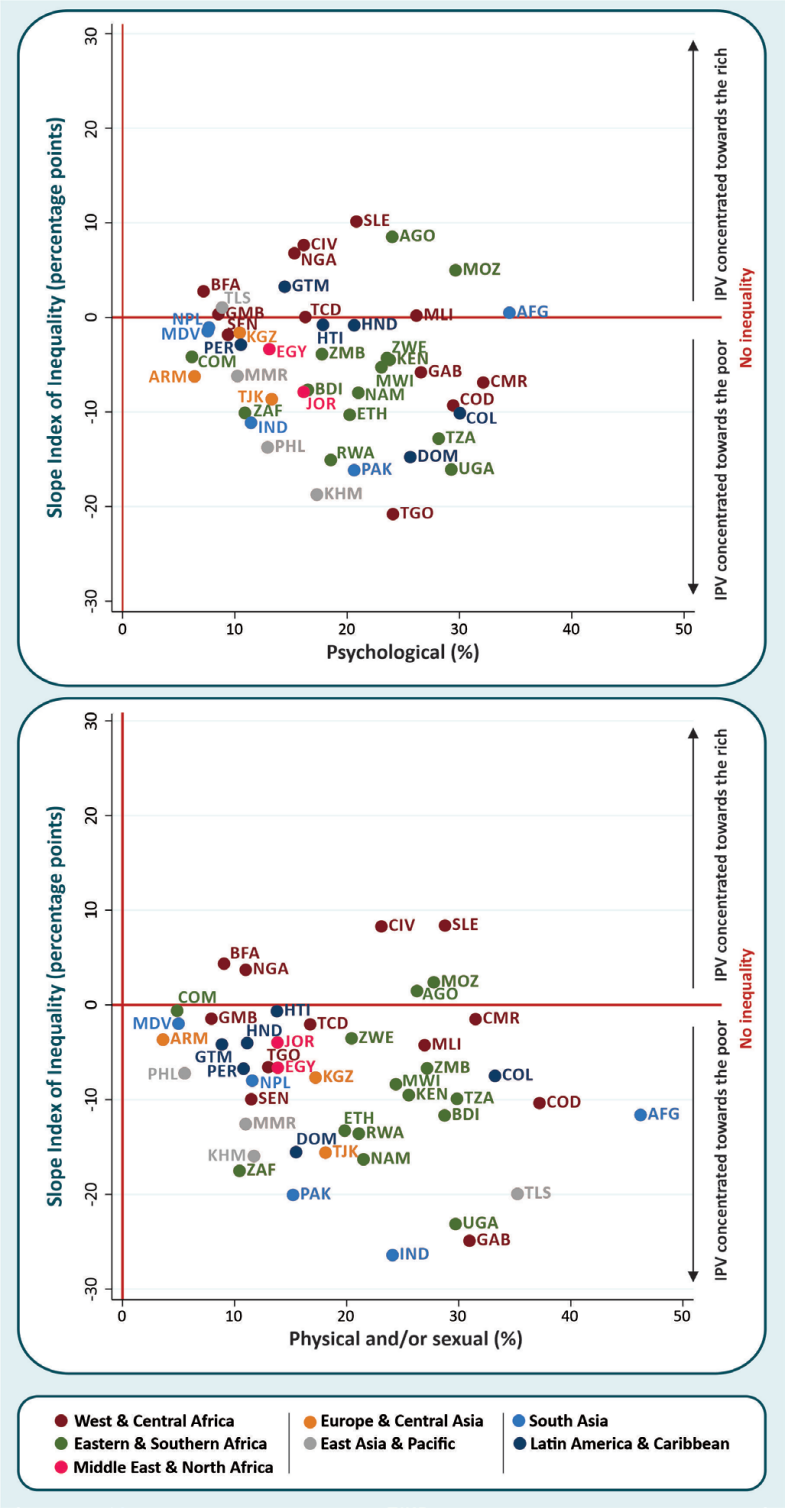
Absolute wealth inequality × national prevalence of recent psychological and physical and/or sexual IPV in each country. IPV, intimate partner violence.

Figure 1 presents a scatter plot of the SII against the prevalence of IPV in each country. There is no clear association between psychological IPV levels and absolute wealth inequality (SII). For physical and/or sexual IPV, high levels of IPV combined with high levels of negative wealth inequalities (with the poorest women faring worst) are observed in several of the countries. In India, Uganda and Gabon, IPV levels above 20% combined with inequalities of more than 20 percentage points were found. Overall, no regional patterns were observed except that similar IPV prevalence with varying inequalities can be observed for Eastern and Southern Africa countries (highlighted in green colour).

**Figure 2:**
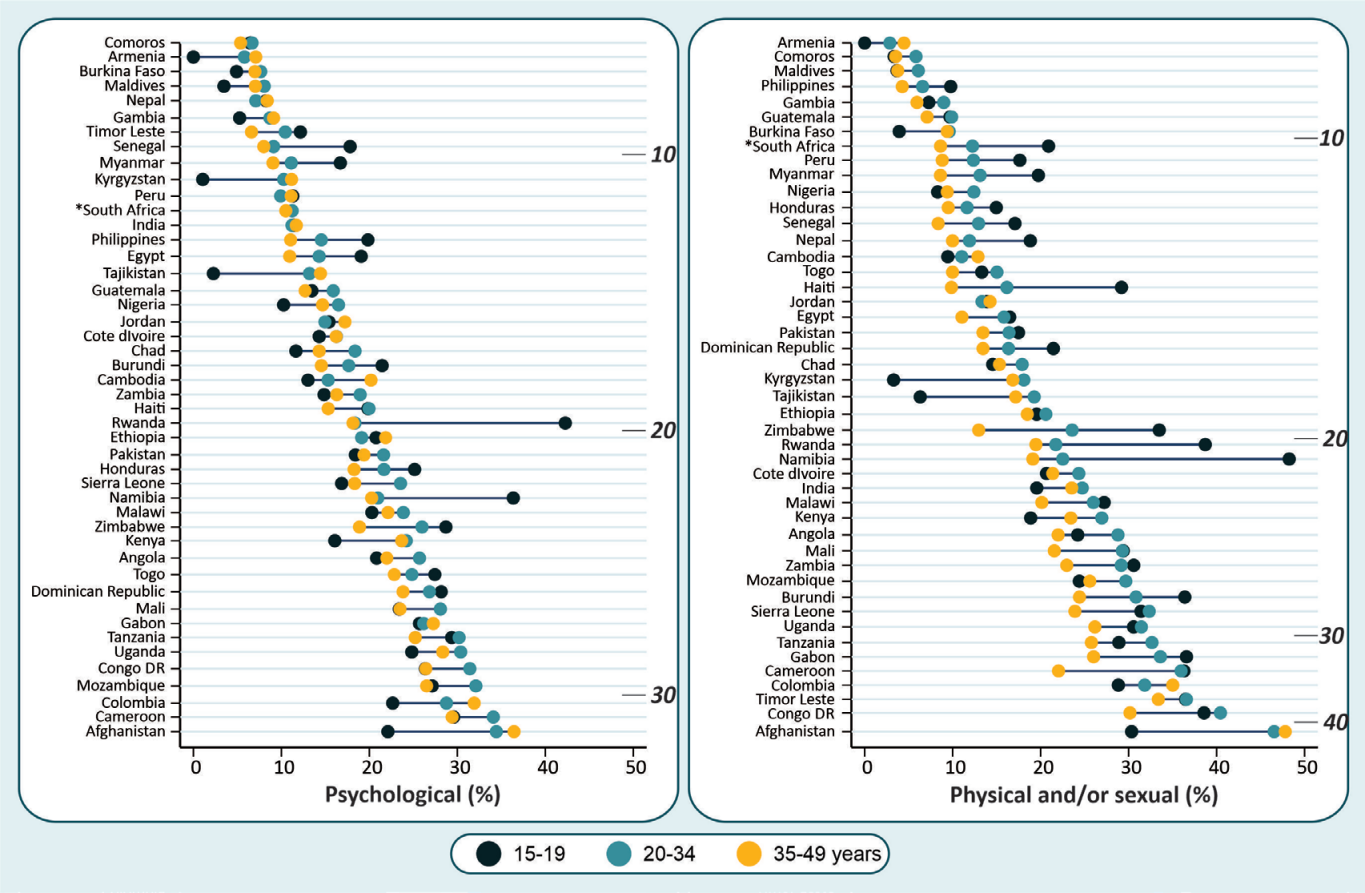
Prevalence of recent psychological and physical and/or sexual IPV according to women’s age in each country. The depth marks indicate the national prevalence of IPV. IPV, intimate partner violence.

Figure 2 shows IPV prevalence by woman’s age groups. Overall, higher levels of IPV were found among younger women for both IPV indicators. In this context, three distinct patterns can be observed. In the first one, a much higher prevalence is observed among adolescents (15–19 years) in comparison with the other age groups. Examples of this pattern include Rwanda, Namibia and Senegal (for psychological IPV). The second pattern was of decreasing physical and/or sexual IPV exposure with woman’s age in a similar spacing across the groups of age. Examples are Philippines, Peru, Myanmar, Honduras, Senegal, Haiti, Dominican Republic, Zimbabwe and Burundi. The third pattern was of much lower levels of physical and/or sexual IPV observed among older women in comparison with the other two age groups. This pattern was observed in countries such as Cameroon, Congo DR, Zambia and Sierra Leone. In a few countries, however, an opposite pattern of lower prevalence among adolescent girls in comparison with the other age groups is observed—within this scenario are Burkina Faso, Kenya, India, Afghanistan, Cambodia, Colombia and all countries from Europe and Central Asia included in our study (Armenia, Kyrgyzstan and Tajikistan). In Afghanistan, as an example, about 50% of the women aged 35–49 years reported having experienced physical or sexual IPV compared with about 30% of the adolescent girls (15–19 years).

Ethiopia and Jordan are example of countries in which no particular vulnerable group emerged (similar IPV estimates were observed across all age groups). For psychological IPV, countries such as Comoros, Nepal, Peru and India also presented no substantial inequalities.

In some countries, varying patterns were observed depending on the nature of the IPV. In Peru, for example, the prevalence of psychological IPV did not vary by age group but the prevalence of physical and/or sexual IPV gradually decreased with age. A similar situation can be observed for Gabon, where no particularly vulnerable group was found for psychological IPV; however, adolescent girls were particularly exposed to physical and/or sexual IPV. Detailed statistics can be found in the online supplementary table S2.

**Figure 3:**
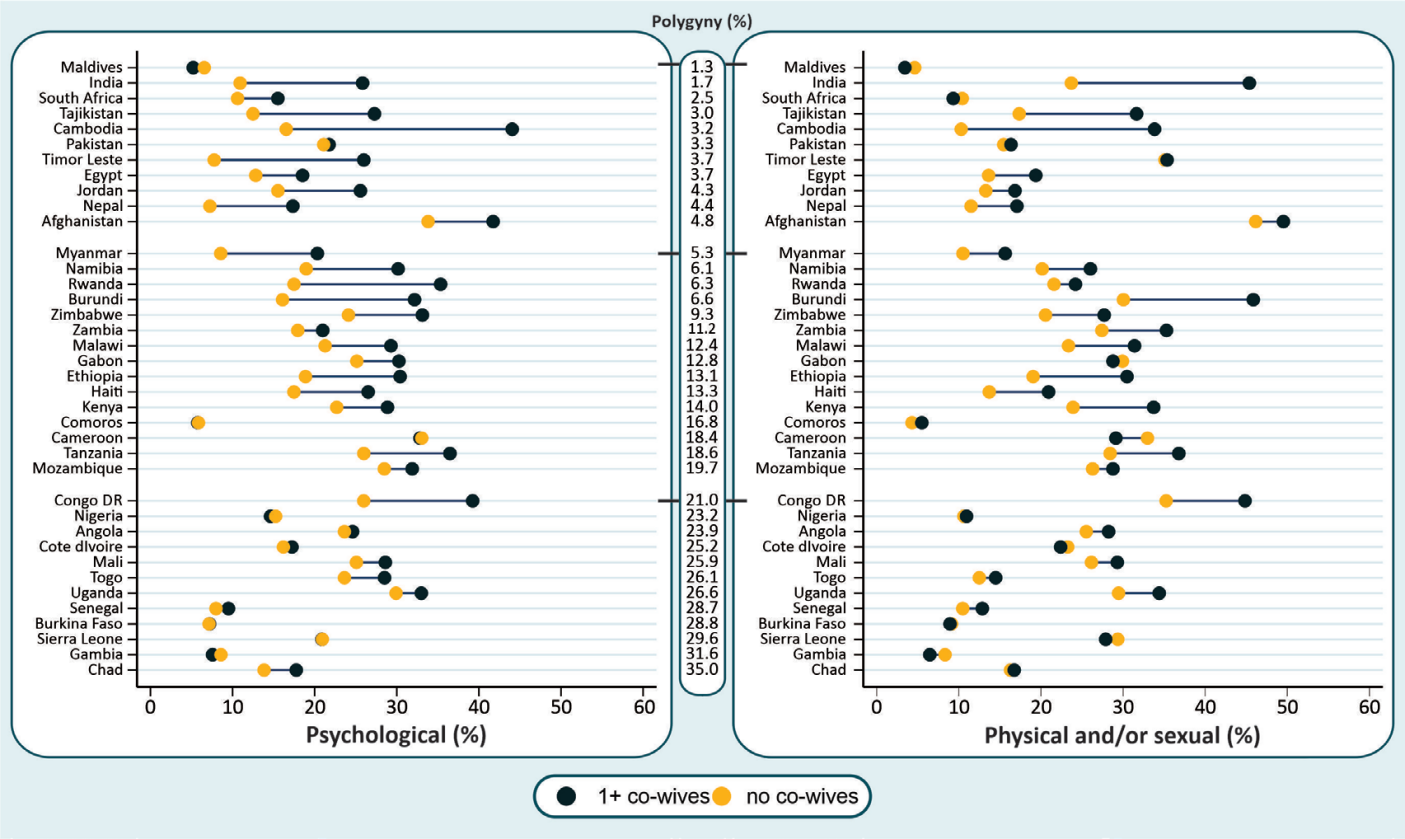
Prevalence of recent psychological and physical and/or sexual IPV according to the polygyny status of the partnership in each country (countries ordered by polygyny prevalence). IPV, intimate partner violence.

Figure 3 presents IPV estimates by polygyny status of the relationship (countries are ordered by their polygyny prevalence). Among the countries studied, polygyny rates varied from 1.3% in the Maldives to 53.9% in Chad. The highest proportions were found in West and Central Africa, with 9 of the 12 countries studied presenting polygyny rates greater than 30%. In countries for which IPV prevalence gaps exist between polygynous and non-polygynous relationships, IPV exposure was consistently higher among women whose partner had multiple cowives for both IPV indicators. Wider gaps tend to be observed for countries in which the proportion of polygyny is below 20%. In this context, the largest gaps for both IPV indicators were found for Cambodia, with the IPV prevalence being more than 20 percentage points higher among women with at least one cowife. However, differences tend to be smaller or null for countries where polygyny rates were above 20%. Detailed statistics can be found in the online supplementary table S3.

**Figure 4:**
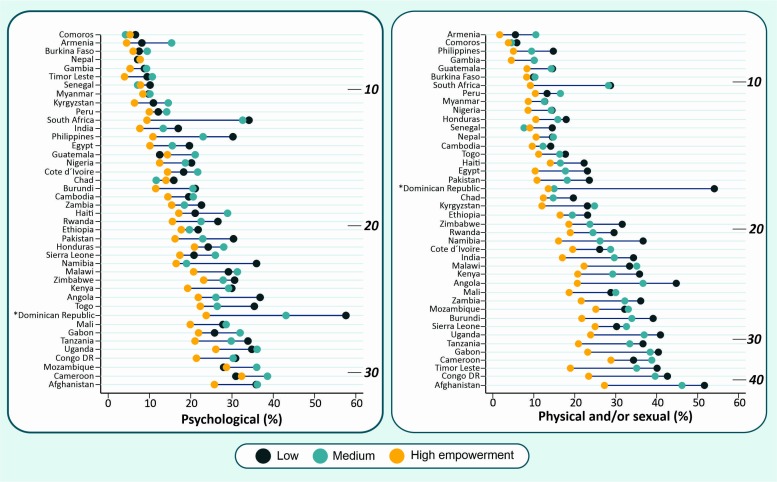
Prevalence of recent psychological and physical and/or sexual IPV by women’s empowerment level according to the SWPER (attitude to violence domain). The depth marks indicate the National prevalence of IPV. IPV, intimate partner violence; SWPER, Survey-based Women’s emPowERment.

IPV levels were consistently lower among more empowered women (figure 4), and this pattern tended to be even more pronounced for physical and/or sexual IPV if compared with psychological IPV. In many countries, there was a clear pattern showing that highly empowered women present much lower IPV experience than the medium or low empowered ones. South Africa and Dominican Republic are two examples where this pattern is very clear, and the differences are huge. However, interpretation of the results from Dominican Republic need caution as the low empowerment group have a small sample size in this country (online supplementary table S4). For physical and/or sexual IPV, these gaps tend to be even higher for countries with national prevalence above 20%. In Afghanistan (the country with the highest IPV prevalence), physical and/or sexual IPV exposure among highly empowered women was more than 20 percentage points lower compared with women in the low empowerment group (27.9% vs 51.3%). For psychological IPV, Guatemala, Mozambique and Cameroon are exceptions of this pattern, presenting higher prevalence among women classified as medium empowerment level as compared with women pertaining to the high and low empowerment groups (which presented similar prevalence).

In the online supplementary table S5, we present IPV levels by area of residence. In most countries, no particularly vulnerable group of women can be identified. For countries where significant urban–rural gaps are observed, however, women living in rural areas tend to present greater IPV exposure. This was particularly evident for physical and/or sexual IPV. In Burundi, for example, the prevalence of physical and/or sexual IPV among rural women was almost two times greater than the prevalence among women living in the urban area (15.0% vs 29.4%). Sierra Leone is among the few cases where the opposite pattern is observed, with urban women being more exposed to both types of IPV as compared with rural women (26.9% vs 18.2% for psychological IPV; 32.2% vs 27% for physical and/or sexual IPV).

## Discussion

We present evidence on the extent of current psychological, physical and sexual IPV and related inequalities using national survey data from 46 LMICs that used similar research design and methods to assess IPV. The findings make it clear that IPV against women is widespread and that inequalities in prevalence, both between and within countries, can be immense. National prevalence of psychological IPV varied from 6.4% in Comoros (Easter and Southern Africa) to 34.4% in Afghanistan (South Asia), while physical and/or sexual IPV varied from 3.5% in Armenia (Europe and Central Asia) to 46% in Afghanistan. The disaggregated analyses revealed prevalence gaps greater than 20 percentage points between some of the population subgroups. Poorer, younger and less empowered women were particularly vulnerable to experience IPV in most LMICs as well as women in polygynous families and those living in rural areas.

Despite the high consistency in the most vulnerable groups of women identified in our analyses, variations from the overall patterns were observed in some countries. For example, while in most countries younger women were particularly vulnerable to IPV exposure, in a few countries such as Afghanistan and Kyrgyzstan, adolescent girls presented a lower IPV prevalence in comparison with women from the other two age groups (aged 20–49 years). The country-specific particularities should therefore be carefully considered for policy programming at the national level.

Nevertheless, the overall patterns observed from the disaggregated analyses clearly points to the relevant intersections between IPV occurrence with poverty and other gender inequality manifestations. Although the intersection between poverty and IPV may be obvious and has been extensively reported in the literature,^26–28^ it is surprising how extreme the patterning by wealth is for some of the countries studied. In India, for example, prevalence gaps between the richest and poor groups of women exceeded 20 percentage points for physical and/or sexual IPV (12.6% in the richest wealth quintile vs 35.3% in the poorest wealth quintile). Our findings also showed that high levels of empowerment were consistently linked to lower IPV exposure. Though the causes of IPV are complex, the role of gender inequality in fostering IPV is well accepted and documented in the literature,^28^ particularly in the context of LMICs where women may experience severely restricted social and economic opportunities relative to men.^29^ More empowered women generally have more control over their own lives and environments and, therefore, a lower probability of suffering from recent IPV as those who suffered abuse may be more likely to seek help. At the same time, women experiencing abuse may also have a greater likelihood of endorsing abuse. This could stem from the fact that repeated abuse may diminish a woman’s self-esteem and thereby increase her propensity to blame herself for whatever reason is triggering IPV (eg, burning the food). Low empowerment may also reflect strong community gender norms that support wife beating.^30^ From either side, our results support the potential effectiveness of interventions that promote women’s empowerment by addressing norms that justify wife beating for IPV reduction.^31^

The strong link between polygyny and violence against women found in the present study have also been reported in the literature.^32^ Explanations for increased IPV in polygynous families have been based on the fact that first wives and their children are often neglected in comparison with the subsequent wives. Second or third wives, however, can be at increased risk of experiencing IPV in cultures where polygyny is illegal but continues to be practised because only the first wife is recognised and protected by the law. In our analyses, we observed varying patterns of inequalities depending on the polygyny prevalence of the country. The wider gaps in IPV occurrence between polygynous and non-polygynous relationships were found in countries where polygyny is not so common. In most of such countries polygyny is not legal,^33^ and this and other drivers of this situation need further investigation.

While the disaggregated analyses can indicate which subgroups of the population are at higher risk and therefore most in need of interventions, the results presented here need to be interpreted in light of the ecological nature of the analyses performed that are not suitable to stablish causal links between IPV exposure and the stratifiers chosen to investigate inequalities. Given the cross-sectional nature of the DHS, these surveys are more useful for surveillance purposes than aetiological analyses. It is also important to recognise that although the factors chosen for data disaggregation provide an important appraisal of the most vulnerable groups of women in the context of LMICs, there are certainly other relevant risk factors that determine women’s vulnerability to IPV exposure that were not explored in our study (eg, women living with disability and transgender women). In addition, while some of the countries included in these data have legal recognition of same-gender marriage, legal gender transition and/or a legally recognised third gender, DHS data are generally collected with an assumption that respondents are cis-women partnered with cis-men and therefore these findings do not account for other relationships and may misclassify some respondents. As far as we are aware, the DHS also do not offer adaptations in data collection methods for women with disabilities and, therefore, this group may be under-represented in our data. This would result in an underestimate of IPV levels since disability has been linked to a higher risk of experiencing IPV among women.

Some caution should be exercised while interpreting the differences in the overall levels of IPV since there will ways be some women who will not disclose information on IPV. Thus, even though the prevalence estimates compiled here allow comparisons across settings, which is unquestionably valuable, they should all probably be considered low-end estimates.^34^ Moreover, the level of under-reporting is likely to vary with respondent’s characteristics as well as cultural and social norms that underlie the acceptance of violence in each setting.^35^ In Burkina Faso and Gambia, for example, relatively low levels of current IPV were observed in comparison with the estimates found in some of the neighbouring countries from the same region. However, very high levels of harmful practices such as female genital mutilation have been described in these countries (prevalence above 70%, with both countries and among the top 10 in the world ranking), which need to be considered in violence against women response efforts.^36^

Despite the inherent limitations of the self-reported data and the implementation of a violence module in a broad health questionnaire, the DHS has been incorporating the best approaches to researching violence against women in an ethically responsible way with act-based standardised questions based on constructs that have been validated. Additionally, the IPV indicators estimated presented a moderate correlation with the Gender Inequality Index,^37^ which measures gender inequalities in three relevant aspects of human development at the country level—reproductive health, empowerment and labour market participation (Person’s correlation 0.44 and 0.45 for physical or sexual IPV and psychological IPV, respectively).

The recognition that all efforts towards achieving the SDGs will be limited without tackling violence against women as a central element of gender inequality creates a unique opportunity to strengthen the investment in and capacity to implement evidence-based strategies. With the rapid increase in the collection of population data on women’s exposure to IPV and other forms of violence, the establishment of proper baselines using reliable data on prevalence is essential for future monitoring on leaving no one behind. The present study advances the current knowledge by providing a global panorama of the prevalence of different forms of IPV across LMICs, helping the identification of the most vulnerable groups of women for which interventions should be prioritised in each country. The monitoring of progress towards the elimination of violence against women and girls will require efforts for routine data collection using standardised and accurate methodologies. These will be essential in order to assess the real impact of prevention and response strategies that have been implemented in the context of LMICs over time. The SDG 5 on gender equality and empowerment of all women and girls includes the elimination of IPV as a target, but it is likely that to tackle IPV we need to empower women first so that they feel like they are entitled to a life free of violence.
