# Supplementary material for: Intimate partner violence in 46 low-income and middle-income countries: an appraisal of the most vulnerable groups of women using national health surveys
Source: BMJ Glob Health. 2020 Jan 26;5(1):e002208. doi: 10.1136/bmjgh-2019-002208 (PMC7042580; doi:10.1136/bmjgh-2019-002208)
Supplement: Supplementary data [file bmjgh-2019-002208supp001.pdf]

S1 Table 1. IPV prevalence according to wealth quintiles and absolute inequalities (SII) by country.

Country	Year	Psychological IPV						Physical and/or sexual IPV					
		Q1 (poorer)	Q2	Q3	Q4	Q5 (richest)	Absolute inequaltity SII (95% CI)*	Q1 (poorer)	Q2	Q3	Q4	Q5 (richest)	Absolute inequality SII (95% CI)*
**West & Central Africa**													
Burkina Faso	2010	7.4	6.8	5.2	6.5	10.2	2.8 (-0.7 to 6.2)	6.8	9.5	8.0	10.0	11.0	**4.4 (2.0 to 6.7)**
Cameroon	2011	33.6	35.0	33.6	29.6	29.6	-6.9 (-14.5 to 0.7)	31.1	32.6	34.0	28.1	31.8	-1.5 (-7.7 to 4.7)
Chad	2014	15.6	16.0	18.2	16.0	15.5	0.0 (-6.0 to 6.0)	15.7	18.3	19.2	14.8	15.2	-2.1 (-7.3 to 3.2)
Congo DR	2013	32.2	32.2	28.9	29.4	24.2	**-9.3 (-17.1 to -1.6)**	41.3	37.1	39.1	36.1	30.8	**-10.4 (-18.1 to -2.6)**
Cote d'Ivoire	2011	12.8	14.6	16.7	18.2	18.6	**7.6 (2.2 to 13.1)**	19.8	20.4	24.4	27.6	23.8	**8.3 (1.9 to 14.7)**
Gabon	2012	28.4	29.8	24.4	26.5	24.2	-5.8 (-14.8 to 3.2)	39.9	40.6	27.4	27.9	20.5	**-24.9 (-32.2 to -17.6)**
Gambia	2013	8.5	9.3	6.7	9.7	8.5	0.3 (-4.8 to 5.5)	8.9	6.6	8.1	9.4	6.3	-1.5 (-6.8 to 3.9)
Mali	2012	24.9	26.7	28.8	24.7	26.0	0.2 (-8.3 to 8.7)	26.1	30.9	28.1	25.2	24.5	-4.3 (-12.1 to 3.6)
Nigeria	2013	9.8	16.8	18.5	15.7	16.4	**6.8 (2.4 to 11.1)**	7.0	11.9	14.1	11.8	10.5	**3.7 (1.0 to 6.5)**
Senegal	2017	11.0	7.8	10.9	7.6	9.3	-1.8 (-7.8 to 4.1)	16.9	9.2	12.9	10.0	6.9	**-10.0 (-15.6 to -4.4)**
Sierra Leone	2013	18.9	18.4	18.4	19.8	28.1	**10.1 (2.3 to 18.0)**	25.7	28.1	28.3	29.0	33.8	**8.3 (0.3 to 16.4)**
Togo	2013	32.8	27.0	25.3	22.6	14.2	**-20.8 (-26.6 to -15.0)**	17.6	12.3	12.8	13.3	9.9	**-6.6 (-10.4 to -2.8)**
**Eastern & Southern Africa**													
Angola	2015	21.3	21.2	23.5	27.6	26.7	**8.5 (0.2 to 16.8)**	25.5	24.6	28.3	28.0	24.8	1.5 (-5.6 to 8.6)
Burundi	2016	19.0	17.7	16.9	15.6	12.3	**-7.7 (-11.4 to -3.9)**	30.8	31.9	30.9	28.5	18.8	**-11.7 (-16.0 to -7.4)**
Comoros	2012	6.9	7.7	6.4	7.1	3.1	-4.2 (-8.8 to 0.5)	3.9	5.9	5.1	6.3	2.9	-0.6 (-4.7 to 3.5)
Ethiopia	2016	21.5	23.6	23.0	20.8	13.1	**-10.3 (-17.3 to -3.3)**	23.4	22.6	22.2	19.4	11.8	**-13.2 (-19.64 to -6.9)**
Kenya	2014	23.5	25.5	26.5	23.3	20.6	-4.5 (-10.7 to 1.7)	27.5	29.7	27.3	22.6	21.6	**-9.5 (-16.2 to -2.8)**
Malawi	2015	24.1	25.0	22.7	23.9	19.6	-5.3 (-10.6 to 0.1)	26.7	26.3	24.5	24.5	19.0	**-8.4 (-13.7 to -3.1)**
Mozambique	2011	29.5	25.2	30.7	31.7	31.2	5.0 (-1.3 to 11.2)	28.2	24.9	28.4	29.2	28.4	2.4 (-3.2 to 8.0)
Namibia	2013	25.4	18.9	21.5	25.3	15.5	-8.0 (-18.8 to 2.9)	26.6	25.6	21.2	25.8	11.9	**-16.3 (-25.7 to -6.9)**
Rwanda	2014	24.7	19.4	20.1	16.4	11.5	**-15.1 (-21.8 to -8.4)**	27.9	22.0	18.4	19.9	15.7	**-13.6 (-20.4 to -6.7)**

South Africa	2016	15.1	14.5	9.7	7.6	8.3	**-10.1 (-16.4 to -3.8)**	17.9	13.6	9.8	8.4	3.2	**-17.5 (-23.4 to -11.6)**
Tanzania	2015	33.9	29.8	29.2	25.9	23.0	**-12.8 (-19.8 to -5.9)**	33.6	30.8	32.3	28.1	25.0	**-9.9 (-15.0 to -4.8)**
Uganda	2016	35.0	30.9	31.6	30.3	19.6	**-16.1 (-20.5 to -11.7)**	36.6	34.4	33.5	28.0	16.9	**-23.1 (-27.2 to -19.0)**
Zambia	2013	17.3	19.4	20.0	18.1	13.9	-3.9 (-7.9 to 0.3)	27.4	28.8	31.1	27.2	20.5	**-6.7 (-11.0 to -2.4)**
Zimbabwe	2015	25.4	23.0	23.2	26.4	19.5	-4.3 (-9.3 to 0.7)	20.9	21.4	18.6	24.8	15.2	-3.5 (-8.0 to 0.9)
**Middle East & North Africa**													
Egypt	2014	15.1	12.1	13.6	13.9	10.6	-3.4 (-7.0 to 0.3)	16.3	14.7	14.6	14.0	9.8	**-6.6 (-10.1 to -3.1)**
Jordan	2017	19.1	19.0	13.4	16.1	12.5	**-7.9 (-13.5 to -2.2)**	15.8	15.4	11.8	13.2	13.0	-4.0 (-9.3 to 1.4)
**Europe & Central Asia**													
Armenia	2015	9.8	6.9	7.1	3.8	5.0	**-6.2 (-10.7 to -1.8)**	6.3	2.7	4.6	1.8	3.0	**-3.7 (-7.0 to -0.4)**
Kyrgyzstan	2012	8.1	11.9	13.5	11.3	7.5	-1.6 (-5.9 to 2.6)	19.5	18.6	19.6	15.8	13.4	**-7.7 (-12.5 to -2.8)**
Tajikistan	2017	15.9	16.4	12.4	12.3	9.7	**-8.6 (-13.9 to -3.4)**	23.6	23.2	15.9	15.8	12.7	**-15.6 (-22.36 to -8.9)**
**South Asia**													
Afghanistan	2015	32.9	34.5	36.9	34.3	33.5	0.5 (-7.3 to 8.3)	48.3	47.1	52.2	46.4	36.8	**-11.6 (-19.7 to -3.5)**
India	2015	16.0	13.7	12.1	9.9	6.7	**-11.1 (-12.7 to -9.6)**	35.3	29.2	25.4	20.5	12.6	**-26.4 (-28.5 to -24.4)**
Maldives	2016	9.2	6.7	6.3	10.0	5.9	-1.5(-6.8 to 3.9)	5.5	5.6	5.2	4.0	4.4	-2.0 (-5.6 to 1.7)
Nepal	2016	7.9	6.8	9.7	7.3	6.6	-1.1 (-5.4 to 3.3)	13.0	14.0	12.6	11.8	6.1	**-8.0 (-12.4 to -3.6)**
Pakistan	2017	22.9	27.9	22.5	18.8	12.5	**-16.2 (-24.4 to -7.9)**	21.2	21.2	15.5	11.1	7.3	**-20.1 (-27.6 to -12.5)**
**East Asia & Pacific**													
Cambodia	2014	23.6	22.8	16.4	15.5	9.0	**-18.7 (-25.4 to -12.1)**	18.9	13.2	11.5	8.5	5.9	**-16.0 (-22.2 to -9.7)**
Myanmar	2015	13.2	12.3	7.4	8.7	9.1	**-6.2 (-11.3 to -1.1)**	16.4	12.2	9.9	7.9	6.6	**-12.6 (-17.3 to -7.8)**
Philippines	2017	17.4	16.8	14.3	8.4	7.7	**-13.7 (-17.2 to -10.3)**	8.3	7.0	4.8	5.0	2.0	**-7.2 (-9.3 to -5.1)**
Timor Leste	2016	7.6	9.1	9.4	10.0	8.2	1.0 (-3.6 to 5.7)	40.9	40.3	36.7	33.1	23.2	**-19.9 (-27.6 to -12.3)**
**Latin America & Caribbean**													
Colombia	2015	30.5	34.1	32.6	29.1	22.7	**-10.1(-13.8 to -6.5)**	31.6	38.3	35.5	33.1	26.5	**-7.5 (-11.5 to -3.5)**
Dominican Republic	2013	31.3	28.0	27.7	21.7	19.1	**-14.8 (-20.8 to -8.8)**	20.9	19.2	15.4	12.7	8.1	**-15.5 (-19.7 to -11.4)**
Guatemala	2014	11.8	13.6	16.5	15.8	14.1	3.2 (-0.6 to 7.1)	10.4	9.6	9.4	8.4	6.8	**-4.2 (-7.3 to -1.0)**
Haiti	2016	18.1	16.9	16.9	21.8	14.7	-0.8 (-6.3 to 4.7)	13.4	12.6	15.4	16.2	10.8	-0.7 (-5.4 to 4.0)

Honduras	2011	18.4	21.7	23.1	21.1	18.3	-0.8 (-4.1 to 2.4)	11.1	12.7	13.0	10.2	8.3	**-4.0 (-6.4 to -1.6)**
Peru	2016	9.5	12.3	12.5	10.0	7.5	**-2.9 (-5.2 to -0.6)**	11.1	13.3	12.7	10.4	5.2	**-6.7 (-8.9 to -4.6)**

*Estimates in bold text refer to statistically significant SII estimates

S2 Table. IPV prevalence by woman´s age group in each country.

Country	Age (years)	N (%)	Psychological IPV % (95% CI)	Psychological IPV p value*	Physical and/or Sexual IPV % (95% CI)	Physical and/or Sexual IPV p value*
**West & Central Africa**						
Burkina Faso (2010)	15-19	795 (7.9)	4.9 (3.2; 7.4)	0.106	4.7 (3.0; 7.2)	0.003
	20-34	6041 (60.4)	7.7 (6.7; 8.7)		9.6 (8.6; 10.7)	
	35-49	3173 (31.7)	6.7 (5.8; 8.4)		9.7 (8.3; 11.2)	
Cameroon (2011)	15-19	309 (7.7)	29.6 (23.6; 36.4)	0.063	36.1 (29.6; 43.2)	<0.001
	20-34	2387 (59.6)	34.1 (31.7; 36.6)		37.0 (34.4; 39.7)	
	35-49	1310 (32.7)	29.4 (26.3; 32.7)		21.0 (18.3; 24.1)	
Chad (2014)	15-19	395 (10.4)	11.7 (8.1; 16.4)	0.016	14.5 (10.6; 19.7)	0.085
	20-34	2214 (58.1)	18.4 (16.1; 20.9)		19.1 (16.7; 21.7)	
	35-49	1205 (31.6)	14.3 (11.7; 17.4)		15.5 (12.8; 18.7)	
Congo DR (2013)	15-19	419 (7.4)	26.4 (20.2; 33.6)	0.033	36.4 (29.2; 44.2)	<0.001
	20-34	3474 (61.0)	31.4 (28.1; 35.0)		40.8 (37.7; 43.9)	
	35-49	1798 (31.6)	26.4 (23.4; 29.6)		29.3 (25.7; 33.2)	
Cote d'Ivoire (2011)	15-19	312 (6.2)	14.3 (9.8; 20.4)	0.832	20.0 (15.1; 26.0)	0.457
	20-34	2972 (59.2)	16.3 (14.1; 18.7)		23.9 (21.7; 26.2)	
	35-49	1734 (34.6)	16.2 (13.6; 19.2)		22.3 (19.4; 25.5)	
Gabon (2012)	15-19	204 (4.9)	25.7 (15.4; 39.7)	0.902	40.3 (30.7; 50.7)	0.005
	20-34	2227 (53.7)	26.2 (23.1; 29.5)		33.9 (30.4; 37.7)	
	35-49	1716 (41.4)	27.3 (23.0; 32.0)		26.1 (21.5; 31.2)	
Gambia (2013)	15-19	269 (7.6)	5.3 (2.3; 9.4)	0.272	5.0 (2.7; 9.0)	0.016
	20-34	2158 (60.9)	8.7 (6.9; 11.0)		8.7 (6.8; 11.0)	
	35-49	1115 (31.5)	9.1 (6.6; 12.4)		5.4 (3.7; 7.8)	
Mali (2012)	15-19	277 (8.9)	23.4 (18.1; 29.8)	0.060	29.3 (22.8; 36.8)	0.004
	20-34	1918 (61.5)	28.1 (25.2; 31.1)		28.8 (25.9; 31.9)	
	35-49	925 (29.7)	23.5 (19.9; 27.4)		21.8 (18.1; 26.0)	
Nigeria (2013)	15-19	1667 (7.5)	10.3 (8.1; 12.8)	<0.001	8.9 (7.3; 10.8)	<0.001
	20-34	12399 (55.6)	16.5 (15.3; 17.7)		12.4 (11.4; 13.4)	
	35-49	8239 (36.9)	14.7 (13.5; 16.0)		9.4 (8.5; 10.4)	
Senegal (2017)	15-19	201 (7.6)	17.8 (9.9; 30.1)	0.028	18.9 (10.9; 30.8)	0.019
	20-34	1479 (55.6)	9.1 (7.2; 11.5)		13.4 (11.0; 16.3)	
	35-49	980 (36.8)	8.0 (6.0; 10.6)		8.9 (6.8; 11.5)	
Sierra Leone (2013)	15-19	239 (5.5)	16.9 (11.8; 23.6)	0.008	30.5 (23.0; 39.2)	<0.001
	20-34	2275 (52.7)	23.5 (20.8; 26.6)		33.1 (30.3; 35.9)	
	35-49	1801 (41.7)	18.3 (15.6; 21.4)		23.4 (20.3; 26.8)	
Togo (2013)	15-19	175 (3.3)	27.5 (20.3; 36.0)	0.253	13.2 (8.8; 19.5)	<0.001
	20-34	3072 (57.1)	24.8 (22.7; 27.1)		14.8 (13.3; 16.4)	
	35-49	2129 (39.6)	22.8 (20.3; 25.5)		10.0 (8.6; 11.6)	
**Eastern & Southern Africa**						
Angola (2015)	15-19	619 (8.1)	20.8 (16.2; 26.3)	0.032	24.3 (19.8; 29.5)	<0.001
	20-34	4649 (60.6)	25.7 (23.2; 28.4)		28.6 (26.3; 31.0)	
	35-49	2401 (31.3)	22.0 (19.0; 25.3)		21.6 (19.1; 24.5)	
Burundi (2016)	15-19	176 (2.4)	21.5 (15.0; 29.8)	0.004	37.8 (29.9; 46.3)	<0.001
	20-34	4588 (62.3)	17.7 (16.2; 19.2)		30.1 (28.5; 31.9)	
	35-49	2602 (35.3)	14.5 (13.0; 16.3)		23.9 (21.9; 26.0)	
Comoros (2012)	15-19	172 (6.8)	6.5 (3.4; 11.9)	0.589	3.7 (1.6; 8.2)	0.159
	20-34	1493 (59.0)	6.7 (5.1; 8.6)		5.7 (4.3; 7.6)	
	35-49	864 (34.2)	5.4 (3.8; 7.5)		3.6 (2.2; 5.8)	

Ethiopia (2016)	15-19	301 (6.4)	20.7	(13.4; 30.8)	0.445	24.3	(16.5; 34.3)	0.323
	20-34	2851 (60.4)	19.1	(16.8; 21.6)		20.3	(18.1; 22.6)	
	35-49	1568 (33.2)	21.8	(18.3; 25.8)		18.1	(15.0; 21.7)	
Kenya (2014)	15-19	153 (3.4)	16.1	(10.2; 24.5)	0.259	20.4	(13.2; 30.1)	0.084
	20-34	2765 (61.2)	24.2	(22.0; 26.6)		26.9	(24.6; 29.4)	
	35-49	1601 (35.4)	23.7	(20.9; 26.7)		23.4	(20.4; 26.6)	
Malawi (2016)	15-19	381 (7.0)	20.3	(15.7; 25.8)	0.362	28.1	(22.6; 34.3)	0.001
	20-34	3443 (63.7)	23.9	(21.9; 26.0)		25.9	(23.9; 28.0)	
	35-49	1582 (29.3)	22.1	(19.5; 24.9)		19.9	(17.4; 22.6)	
Mozambique (2011)	15-19	589 (10.1)	27.1	(23.2; 31.5)	0.002	22.5	(18.9; 26.6)	0.001
	20-34	3322 (57.0)	32.1	(30.0; 34.3)		30.2	(27.9; 32.6)	
	35-49	1913 (32.9)	26.5	(24.1; 29.0)		25.3	(22.8; 27.9)	
Namibia (2013)	15-19	29 (2.2)	36.4	(17.1; 61.3)	0.251	52.0	(29.4; 73.8)	0.001
	20-34	685 (51.4)	21.0	(17.4; 25.0)		21.2	(17.4; 25.6)	
	35-49	619 (46.4)	20.3	(16.5; 24.6)		17.6	(14.2; 21.6)	
Rwanda (2014)	15-19	23 (1.2)	42.3	(24.4; 62.4)	0.030	33.8	(17.1; 55.8)	0.071
	20-34	1173 (61.5)	18.4	(16.1; 20.9)		22.0	(19.3; 24.9)	
	35-49	712 (37.3)	18.1	(15.3; 21.4)		18.3	(15.4; 21.6)	
South Africa (2016)	15-19	16 (0.7)	17.5	(4.7; 47.8)	0.703	21.5	(6.7; 51.2)	0.120
	20-34	1066 (45.3)	11.2	(9.0; 13.9)		11.9	(9.4; 14.8)	
	35-49	1272 (54.0)	10.5	(8.5; 12.9)		9.1	(6.8; 12.0)	
Tanzania (2015)	15-19	466 (6.1)	29.4	(24.2; 35.2)	0.003	30.1	(25.2; 35.5)	<0.001
	20-34	4276 (56.3)	30.2	(28.1; 32.3)		32.7	(30.8; 34.7)	
	35-49	2855 (37.6)	25.2	(23.0; 27.6)		25.1	(22.8; 27.4)	
Uganda (2016)	15-19	531 (7.1)	24.8	(20.7; 29.4)	0.053	30.6	(25.7; 36.1)	0.007
	20-34	4614 (61.2)	30.4	(28.6; 32.2)		31.2	(29.4; 33.2)	
	35-49	2391 (31.7)	28.3	(26.0; 30.8)		26.7	(24.5; 29.0)	
Zambia (2013)	15-19	481 (5.1)	14.9	(11.4; 19.1)	0.016	29.8	(25.0; 35.1)	<0.001
	20-34	5803 (61.6)	19.0	(17.4; 20.6)		28.9	(27.2; 30.7)	
	35-49	3132 (33.3)	16.3	(14.7; 18.1)		22.3	(20.0; 24.7)	
Zimbabwe (2015)	15-19	310 (5.3)	28.7	(23.1; 35.0)	<0.001	31.5	(24.8; 39.1)	<0.001
	20-34	3458 (59.6)	26.0	(23.9; 28.1)		23.1	(21.2; 25.1)	
	35-49	2032 (35.0)	18.9	(16.4; 21.7)		12.8	(10.9; 14.8)	
**Middle East & North Africa**								
Egypt (2014)	15-19	211 (3.2)	19.1	(12.8; 27.4)	0.002	16.5	(11.0; 24.1)	<0.001
	20-34	3663 (54.7)	14.3	(12.9; 15.8)		16.0	(14.6; 17.5)	
	35-49	2819 (42.1)	10.9	(9.5; 12.5)		11.1	(9.7; 12.7)	
Jordan (2017)	15-19	173 (2.5)	15.4	(8.6; 26.1)	0.285	14.5	(8.2; 24.2)	0.927
	20-34	3228 (47.1)	15.0	(13.1; 17.0)		13.5	(11.6; 15.8)	
	35-49	3451 (50.4)	17.2	(15.1; 19.6)		14.0	(11.9; 16.4)	
**Europe & Central Asia**								
Armenia (2015)	15-19	26 (0.7)	0.0	(0; 0)	0.211	0.0	(0; 0)	0.072
	20-34	1816 (51.3)	5.8	(4.7; 7.2)		2.7	(2.0; 3.7)	
	35-49	1698 (48.0)	7.1	(5.6; 8.9)		4.3	(3.2; 5.7)	
Kyrgyzstan (2012)	15-19	108 (2.2)	1.1	(0.3; 4.4)	0.010	2.7	(1.1; 6.7)	0.001
	20-34	2634 (54.5)	10.3	(8.6; 12.3)		17.8	(15.6; 20.4)	
	35-49	2090 (43.3)	11.2	(9.5; 13.0)		17.2	(15.1; 19.5)	
Tajikistan (2017)	15-19	122 (2.3)	2.3	(0.7; 7.0)	0.001	5.6	(2.8; 11.1)	<0.001
	20-34	3022 (56.9)	13.2	(11.6; 15.0)		20.9	(18.4; 23.5)	
	35-49	2169 (40.8)	14.4	(12.1; 17.1)		17.5	(15.0; 20.4)	
**South Asia**								

Afghanistan (2015)	15-19	1060 (5.0)	22.1	(17.4; 27.6)	<0.001	28.7	(24.3; 33.6)	<0.001
	20-34	12099 (56.7)	34.4	(32.5; 36.4)		46.0	(43.6; 48.5)	
	35-49	8165 (38.3)	36.4	(34.2; 38.7)		48.9	(46.5; 51.3)	
India (2015)	15-19	1642 (2.5)	11.4	(9.1; 14.2)	0.5499	17.4	(15.0; 20.2)	<0.001
	20-34	36415 (55.2)	11.2	(10.7; 11.8)		24.3	(23.5; 25.2)	
	35-49	27956 (42.3)	11.7	(11.0; 12.4)		23.4	(22.5; 24.3)	
Maldives (2016)	15-19	28 (0.8)	3.5	(0.46; 22.1)	0.569	3.5	(0.5; 22.1)	0.016
	20-34	1803 (53.2)	8.1	(6.2; 10.5)		6.9	(5.2; 9.1)	
	35-49	1557 (46.0)	7.1	(5.2; 9.5)		3.7	(2.4; 5.6)	
Nepal (2016)	15-19	223 (5.8)	8.3	(4.8; 14.0)	0.537	17.0	(11.4; 24.7)	0.009
	20-34	2170 (56.7)	7.1	(5.9; 8.6)		11.9	(10.2; 13.8)	
	35-49	1433 (37.5)	8.4	(6.4; 10.9)		9.3	(7.6; 11.5)	
Pakistan (2017)	15-19	129 (3.9)	18.4	(11.4; 28.3)	0.493	17.3	(10.1; 27.8)	0.376
	20-34	1769 (53.6)	21.6	(18.5; 25.1)		15.2	(13.0; 17.8)	
	35-49	1405 (42.5)	19.4	(16.5; 22.6)		13.1	(10.8; 15.8)	
**East Asia & the Pacific**								
Cambodia (2014)	15-19	106 (3.0)	13	(6.9; 23.4)	0.015	7.0	(2.7; 17.3)	0.354
	20-34	2011 (57.5)	15.3	(13.4; 17.5)		10.4	(8.6; 12.4)	
	35-49	1382 (39.5)	20.2	(17.2; 23.5)		12.0	(9.9; 14.5)	
Myanmar (2015)	15-19	84 (2.5)	16.7	(8.7; 29.6)	0.0996	21.3	(11.9; 35.2)	0.001
	20-34	1589 (46.4)	11.1	(9.2; 13.4)		12.8	(10.9; 15.1)	
	35-49	1752 (51.2)	9	(7.5; 10.9)		8.8	(7.3; 10.6)	
Philippines (2017)	15-19	303 (2.3)	19.8	(13.7; 27.9)	<0.001	11.1	(6.6; 18.0)	<0.001
	20-34	6506 (49.2)	14.5	(13.2; 16.0)		6.6	(5.7; 7.6)	
	35-49	6406 (48.5)	11.1	(9.9; 12.3)		4.1	(3.5; 4.8)	
Timor Leste (2016)	15-19	90 (2.4)	12.2	(5.7; 24.2)	0.011	37.7	(25.8; 51.2)	0.383
	20-34	2031 (55.0)	10.4	(8.6; 12.7)		35.7	(32.7; 38.8)	
	35-49	1573 (42.6)	6.6	(5.2; 8.4)		33.0	(29.8; 36.3)	
**Latin America & Caribbean**								
Colombia (2015)	15-19	1227 (4.9)	22.7	(19.8; 25.8)	<0.001	28.8	(25.2; 32.8)	0.001
	20-34	11732 (47.2)	28.8	(27.4; 30.2)		31.8	(30.5; 33.2)	
	35-49	11903 (47.9)	31.9	(30.6; 33.3)		35.0	(33.4; 36.7)	
Dominican Republic (2013)	15-19	416 (7.2)	28.2	(22.2; 35.0)	0.162	21.6	(15.7; 29.0)	0.031
	20-34	3029 (52.2)	26.8	(24.5; 29.3)		16.1	(14.3; 17.9)	
	35-49	2358 (40.6)	23.8	(21.2; 26.7)		14.1	(11.9; 16.7)	
Guatemala (2014)	15-19	403 (6.2)	13.5	(9.9; 18.1)	0.028	8.6	(6.0; 12.3)	0.001
	20-34	3719 (57.1)	15.9	(14.4; 17.6)		9.9	(8.6; 11.5)	
	35-49	2390 (36.7)	12.7	(11.1; 14.5)		6.5	(5.4; 7.9)	
Haiti (2016)	15-19	129 (3.0)	19.9	(13.0; 29.0)	0.008	28.0	(20.1; 37.7)	<0.001
	20-34	2279 (52.7)	20.0	(17.7; 22.4)		16.9	(14.6; 19.4)	
	35-49	1914 (44.3)	15.3	(13.2; 17.7)		9.4	(7.7; 11.3)	
Honduras (2011)	15-19	1011 (8.1)	25.1	(21.7; 29.0)	<0.001	16.4	(13.4; 19.8)	<0.001
	20-34	7093 (56.8)	21.7	(20.3; 23.0)		11.5	(10.5; 12.5)	
	35-49	4390 (35.1)	18.3	(16.8; 19.8)		9.0	(8.0; 10.1)	
Peru (2016)	15-19	728 (3.4)	11.3	(8.1; 15.6)	0.180	17.6	(13.7; 22.4)	<0.001
	20-34	12158 (57.6)	9.9	(9.1; 10.8)		12.4	(11.5; 13.3)	
	35-49	8229 (39.0)	11.1	(10.1; 12.3)		8.8	(7.9; 9.9)	

***** p value based on χ^2 test for differences between groups

S3 Table. IPV prevalence by polygyny status of the relationship in each country.

Country	Polygyny status (co-wives)	N (%)	Psychological IPV			Physical and/or Sexual IPV		
			%	(95% CI)	p value*	%	(95% CI)	p value*
**West & Central Africa**								
Burkina Faso (2010)	none	6863 (71.2)	7.2	(6.2; 8.2)	0.943	9.3	(8.4; 10.4)	0.747
	1+	2775 (28.8)	7.2	(6.1; 8.5)		9.1	(7.8; 10.6)	
Cameroon (2011)	none	2780 (81.6)	33.0	(30.8; 35.4)	0.934	33.0	(30.7; 35.4)	0.197
	1+	627 (18.4)	32.8	(28.1; 37.9)		29.6	(25.4; 34.3)	
Chad (2014)	none	2245 (65.0)	13.8	(12.0; 16.0)	0.038	15.8	(13.7; 18.2)	0.226
	1+	1210 (35.0)	17.8	(15.0; 20.9)		18.1	(15.1; 21.6)	
Congo DR (2013)	none	3972 (79.0)	26.0	(23.7; 28.4)	<0.001	34.7	(31.5; 38.0)	<0.001
	1+	1054 (21.0)	39.2	(33.9; 44.9)		43.8	(39.0; 48.6)	
Cote d'Ivoire (2011)	none	3442 (74.8)	16.2	(14.3; 18.3)	0.0584	22.7	(20.7; 24.8)	0.460
	1+	1158 (25.2)	17.2	(13.8; 21.3)		24.3	(20.5; 28.5)	
Gabon (2012)	none	2876 (87.2)	25.1	(22.2; 28.4)	0.217	30.4	(27.2; 33.8)	0.941
	1+	423 (12.8)	30.3	(23.2; 38.4)		30.1	(22.3; 39.2)	
Gambia (2013)	none	2264 (68.4)	8.6	(6.8; 10.8)	0.483	7.4	(5.7; 9.6)	0.520
	1+	1048 (31.6)	7.6	(5.6; 10.2)		6.5	(4.7; 9.1)	
Mali (2012)	none	2259 (74.1)	25.1	(22.3; 28.1)	0.098	25.6	(22.8; 28.7)	0.196
	1+	788 (25.9)	28.6	(24.9; 32.6)		28.4	(24.6; 32.6)	
Nigeria (2013)	none	16005 (76.8)	15.3	(14.2; 16.4)	0.391	10.8	(9.9; 11.7)	0.385
	1+	4846 (23.2)	14.6	(13.1; 16.3)		10.2	(9.1; 11.5)	
Senegal (2017)	none	1782 (71.3)	8.0	(6.3; 10.1)	0.344	10.8	(8.7; 13.2)	0.204
	1+	718 (28.7)	9.5	(7.0; 12.7)		13.2	(10.1; 16.9)	
Sierra Leone (2013)	none	2796 (70.4)	20.9	(18.7; 23.4)	0.983	29.2	(26.6; 31.9)	0.631
	1+	1174 (29.6)	20.9	(18.1; 24.0)		28.1	(24.9; 31.6)	
Togo (2013)	none	3588 (73.9)	23.6	(21.8; 25.6)	0.005	12.0	(10.8; 13.3)	0.060
	1+	1269 (26.1)	28.5	(25.1; 32.2)		14.2	(11.9; 16.9)	
**Eastern & Southern Africa**								
Angola (2015)	none	4963 (76.1)	23.6	(20.8; 26.8)	0.598	25.1	(22.8; 27.6)	0.195
	1+	1561 (23.9)	24.6	(21.4; 28.2)		27.4	(24.3; 30.8)	
Burundi (2016)	none	5947 (93.4)	16.1	(14.9; 17.4)	<0.001	29.5	(28.0; 31.1)	<0.001
	1+	423 (6.6)	32.2	(27.4; 37.3)		44.6	(39.4; 50.0)	
Comoros (2012)	none	1868 (83.2)	5.8	(4.7; 7.2)	0.966	4.0	(3.0; 5.4)	0.198
	1+	376 (16.8)	5.8	(3.3; 9.8)		6.0	(3.5; 10.3)	
Ethiopia (2016)	none	3558 (86.9)	18.9	(16.7; 21.3)	<0.001	18.9	(16.9; 21.1)	0.003
	1+	535 (13.1)	30.4	(22.6; 39.5)		28.8	(22.2; 36.4)	
Kenya (2014)	none	3258 (86.0)	22.7	(20.9; 24.6)	0.022	23.9	(21.9; 26.0)	<0.001
	1+	530 (14.0)	28.9	(24.3; 33.9)		33.9	(29.0; 39.2)	
Malawi (2015)	none	4027 (87.6)	21.3	(19.6; 23.1)	0.001	23.2	(21.3; 25.3)	0.004
	1+	572 (12.4)	29.3	(24.7; 34.3)		30.6	(25.9; 35.7)	
Mozambique (2011)	none	3647 (80.3)	28.5	(26.7; 30.4)	0.120	26.3	(24.4; 28.3)	0.292
	1+	895 (19.7)	31.9	(27.8; 36.2)		28.7	(25.0; 32.6)	
Namibia (2013)	none	943 (94.3)	19.0	(15.9; 22.4)	0.065	18.5	(15.6; 21.9)	0.100
	1+	57 (5.7)	30.2	(18.3; 45.4)		28.9	(18.1; 42.9)	
Rwanda (2014)	none	1515 (93.7)	17.5	(15.5; 19.6)	<0.001	21.3	(18.9; 23.9)	0.834
	1+	102 (6.3)	35.3	(26.1; 45.8)		22.2	(15.1; 31.4)	
South Africa (2016)	none	1887 (97.5)	10.6	(8.9; 12.6)	0.361	10.2	(8.4; 12.4)	0.816
	1+	48 (2.5)	15.5	(6.9; 31.5)		11.4	(4.6; 25.5)	

Tanzania (2015)	none	5229 (81.4)	26.0	(24.2; 27.9)	<0.001	27.7	(25.9; 29.4)	<0.001
	1+	1191 (18.6)	36.5	(31.6; 41.6)		35.8	(31.8; 39.9)	
Uganda (2016)	none	4521 (73.4)	29.9	(28.1; 31.8)	0.055	29.1	(27.2; 31.2)	0.002
	1+	1639 (26.6)	33.0	(30.2; 35.9)		34.2	(31.6; 37.0)	
Zambia (2013)	none	7018 (88.8)	17.9	(16.6; 19.3)	0.059	27.0	(25.5; 28.5)	0.001
	1+	886 (11.2)	21.0	(18.0; 24.3)		33.5	(29.6; 37.7)	
Zimbabwe (2015)	none	4406 (90.8)	24.1	(22.4; 25.9)	0.001	20.0	(18.6; 21.6)	0.013
	1+	449 (9.2)	33.1	(28.0; 38.6)		26.2	(21.4; 31.5)	
**Middle East & North Africa**								
Egypt (2014)	none	6433 (96.3)	12.8	(11.8; 13.9)	0.062	13.6	(12.6; 14.7)	0.006
	1+	246 (3.7)	18.5	(12.7; 26.2)		22.4	(15.7; 30.9)	
Jordan (2017)	none	6177 (95.7)	15.5	(14.0; 17.2)	0.006	13.4	(11.8; 15.1)	0.366
	1+	278 (4.3)	25.6	(18.1; 34.9)		16.3	(10.6; 24.2)	
**Europe & Central Asia**								
Tajikistan (2017)	none	4660 (97.0)	12.5	(10.9; 14.2)	<0.001	18.2	(16.1; 20.5)	<0.001
	1+	142 (3.0)	27.3	(18.5; 38.2)		29.5	(20.4; 40.6)	
**South Asia**								
Afghanistan (2015)	none	19710 (95.2)	33.8	(32.2; 35.5)	0.003	45.7	(43.5; 48.0)	0.032
	1+	991 (4.8)	41.7	(36.4; 47.3)		51.8	(45.9; 57.6)	
India (2015)	none	61619 (98.3)	10.9	(10.5; 11.4)	<0.001	23.3	(22.6; 24.0)	<0.001
	1+	1045 (1.7)	25.8	(21.7; 30.4)		43.9	(39.3; 48.7)	
Maldives (2016)	none	3036 (98.7)	6.6	(5.1; 8.4)	0.785	5.0	(3.9; 6.5)	0.222
	1+	39 (1.3)	5.2	(0.9; 24.2)		2.1	(0.5; 8.7)	
Nepal (2016)	none	3536 (95.6)	7.2	(6.1; 8.5)	<0.001	11.0	(9.8; 12.5)	0.018
	1+	163 (4.4)	17.4	(11.5; 25.3)		17.8	(12.2; 25.4)	
Pakistan (2017)	none	3094 (96.7)	21.1	(18.8; 23.6)	0.908	14.8	(13.1; 16.7)	0.838
	1+	105 (3.3)	21.8	(11.4; 37.5)		15.6	(8.5; 27.0)	
**East Asia & the Pacific**								
Cambodia (2014)	none	3025 (96.8)	16.5	(14.8; 18.5)	<0.001	9.2	(7.9; 10.6)	<0.001
	1+	99 (3.2)	44.1	(31.3; 57.7)		37.5	(24.6; 52.6)	
Myanmar (2015)	none	2958 (94.7)	8.6	(7.4; 10.0)	<0.001	10.4	(9.1; 11.9)	0.153
	1+	165 (5.3)	20.3	(12.8; 30.7)		14.9	(9.4; 22.9)	
Timor Leste (2016)	none	3538 (96.4)	7.8	(6.6; 9.1)	<0.001	34.5	(32.1; 36.9)	0.705
	1+	134 (3.6)	26.0	(14.3; 42.4)		32.3	(21.9; 44.8)	
**Latin America & Caribbean**								
Haiti (2016)	none	3304 (86.8)	17.5	(15.6; 19.5)	<0.001	13.6	(12.0; 15.3)	0.002
	1+	504 (13.2)	26.5	(22.0; 31.6)		20.6	(16.6; 25.3)	

***** p value based on χ^2 test for differences between groups

S4 Table. IPV prevalence by women´s empowerment level in each country.

Country	Empowerment level (SWPER)	N (%)	Psychological IPV			Physical and/or Sexual IPV		
			%	(95% CI)	p value*	%	(95% CI)	p value*
**West & Central Africa**								
Burkina Faso (2010)	low	2343 (24.7)	7.5	(6.1; 9.2)	<0.001	10.5	(8.8; 12.5)	0.009
	medium	1950 (20.5)	9.5	(7.6; 11.7)		10.4	(8.6; 12.5)	
	high	5208 (54.8)	6.1	(5.2; 7.0)		8.1	(7.1; 9.3)	
Cameroon (2011)	low	683 (20.0)	30.9	(26.7; 35.5)	0.016	35.5	(30.5; 40.9)	<0.001
	medium	892 (26.1)	38.6	(34.7; 42.6)		39.2	(34.9; 43.6)	
	high	1839 (53.9)	32.3	(29.7; 35.1)		27.8	(25.1; 30.5)	
Chad (2014)	low	1632 (51.1)	15.9	(13.7; 18.3)	0.186	19.9	(17.2; 22.9)	0.001
	medium	625 (19.6)	11.6	(8.8; 15.3)		13.8	(9.6; 19.3)	
	high	934 (29.3)	14.0	(10.2; 18.9)		12.3	(9.2; 16.4)	
Congo DR (2013)	low	2432 (48.8)	30.9	(27.9; 34.0)	<0.001	41.4	(37.7; 45.2)	<0.001
	medium	1362 (27.3)	30.3	(25.5; 35.5)		39.4	(34.4; 44.6)	
	high	1193 (23.9)	21.4	(18.3; 24.9)		23.5	(19.7; 27.8)	
Cote d'Ivoire (2011)	low	1367 (32.4)	18.3	(15.2; 21.8)	0.003	24.6	(21.3; 28.2)	<0.001
	medium	842 (20.0)	21.7	(17.6; 26.4)		29.5	(24.6; 35.0)	
	high	2004 (47.6)	14.4	(11.9; 17.3)		20.4	(17.6; 23.5)	
Gabon (2012)	low	848 (27.3)	25.8	(20.8; 31.5)	0.005	39.7	(33.6; 46.2)	<0.001
	medium	918 (29.6)	32.0	(26.7; 37.7)		39.7	(33.8; 45.9)	
	high	1340 (43.1)	21.9	(17.6; 26.9)		23.3	(19.8; 27.1)	
Gambia (2013)	low	1053 (34.0)	8.8	(6.3; 12.1)	0.033	8.9	(6.1; 12.6)	<0.001
	medium	931 (30.1)	9.3	(6.8; 12.5)		10.0	(7.7; 13.1)	
	high	1114 (36.0)	5.3	(3.8; 7.5)		3.6	(2.1; 5.9)	
Mali (2012)	low	1668 (54.7)	27.8	(24.8; 31.0)	0.003	27.5	(24.6; 30.7)	<0.001
	medium	721 (23.6)	28.6	(23.4; 34.4)		31.2	(25.4; 37.6)	
	high	661 (21.7)	19.9	(16.0; 24.4)		19.0	(15.3; 23.5)	
Nigeria (2013)	low	4616 (22.6)	20.2	(17.9; 22.6)	<0.001	14.7	(13.1; 16.4)	<0.001
	medium	3502 (17.1)	18.7	(16.7; 20.9)		13.5	(12.0; 15.2)	
	high	12332 (60.3)	12.5	(11.4; 13.6)		8.5	(7.7; 9.4)	
Senegal (2017)	low	1124 (49.4)	10.2	(7.9; 12.9)	0.343	14.7	(11.8; 18.2)	0.004
	medium	232 (10.2)	7.1	(3.7; 13.3)		6.5	(3.8; 11.0)	
	high	920 (40.4)	7.8	(5.7; 10.7)		9.7	(7.4; 12.7)	
Sierra Leone (2013)	low	1879 (48.5)	20.8	(18.5; 23.3)	0.006	29.2	(26.5; 32.1)	0.052
	medium	738 (19.0)	26.0	(20.7; 32.0)		33.4	(27.9; 39.4)	
	high	1259 (32.5)	17.4	(14.5; 20.7)		26.2	(22.6; 30.1)	
Togo (2013)	low	904 (19.1)	35.4	(30.8; 40.4)	<0.001	19.1	(15.8; 22.9)	<0.001
	medium	750 (15.8)	26.4	(22.9; 30.3)		14.9	(12.0; 18.4)	
	high	3089 (65.1)	22.3	(20.1; 24.7)		10.6	(9.4; 11.9)	
**Eastern & Southern Africa**								
Angola (2015)	low	906 (15.0)	36.8	(32.1; 41.8)	<0.001	42.5	(37.8; 47.3)	<0.001
	medium	1006 (16.7)	26.1	(22.5; 30.0)		35.2	(30.8; 39.9)	
	high	4118 (68.3)	21.8	(18.7; 25.4)		20.5	(18.2; 23.1)	
Burundi (2016)	low	2160 (33.8)	21.1	(19.1; 23.2)	<0.001	38.8	(36.3; 41.3)	<0.001
	medium	1741 (27.2)	20.6	(18.2; 23.2)		33.3	(30.7; 36.1)	
	high	2488 (38.9)	11.5	(10.0; 13.2)		21.2	(19.2; 23.2)	
Comoros (2012)	low	401 (19.2)	6.6	(4.0; 10.7)	0.419	5.3	(3.3; 8.6)	0.449
	medium	450 (21.6)	4.2	(2.5; 7.1)		4.8	(2.7; 8.4)	

	high	1234 (59.2)	5.3	(4.0; 7.1)		3.7	(2.6; 5.3)	
Ethiopia (2016)	low	1724 (42.3)	21.8	(18.5; 25.4)	0.180	23.0	(20.1; 26.2)	0.005
	medium	762 (18.7)	19.7	(15.2; 25.1)		18.9	(14.9; 23.7)	
	high	1589 (39.0)	17.7	(14.6; 21.3)		15.8	(13.2; 18.9)	
Kenya (2014)	low	853 (22.5)	29.9	(25.4; 34.9)	<0.001	35.2	(30.6; 40.2)	<0.001
	medium	1023 (27.0)	29.2	(25.6; 33.1)		29.3	(25.6; 33.3)	
	high	1915 (50.5)	19.3	(17.1; 21.6)		20.6	(18.1; 23.4)	
Malawi (2015)	low	268 (6.0)	29.1	(22.4; 36.9)	<0.001	35.0	(27.7; 43.1)	<0.001
	medium	422 (9.4)	31.3	(25.5; 37.8)		32.6	(27.2; 38.5)	
	high	3810 (84.7)	20.7	(18.9; 22.6)		22.1	(20.2; 24.1)	
Mozambique (2011)	low	293 (6.2)	28.0	(21.7; 35.3)	0.002	31.4	(25.1; 38.5)	<0.001
	medium	805 (17.0)	36.0	(31.4; 40.9)		33.6	(29.3; 38.2)	
	high	3640 (76.8)	28.7	(26.7; 30.7)		25.1	(23.1; 27.2)	
Namibia (2013)	low	151 (12.6)	35.9	(26.6; 46.5)	<0.001	36.9	(28.4; 46.4)	<0.001
	medium	219 (18.2)	19.0	(13.2; 26.5)		25.3	(18.3; 33.9)	
	high	833 (69.2)	16.5	(13.5; 20.0)		14.1	(11.4; 17.3)	
Rwanda (2014)	low	271 (16.8)	26.6	(21.1; 32.9)	<0.001	30.6	(23.9; 38.3)	<0.001
	medium	345 (21.4)	22.5	(18.1; 27.7)		23.9	(19.4; 29.0)	
	high	993 (61.7)	15.6	(13.3; 18.2)		18.1	(15.5; 21.0)	
South Africa (2016)	low	32 (1.7)	34.1	(16.3; 58.0)	<0.001	25.5	(9.9; 51.7)	<0.001
	medium	76 (3.9)	32.6	(18.7; 50.5)		30.2	(17.4; 47.2)	
	high	1831 (94.4)	9.4	(7.9; 11.2)		9.0	(7.2; 11.2)	
Tanzania (2015)	low	2569 (39.8)	33.9	(31.0; 36.8)	<0.001	35.9	(33.1; 38.7)	<0.001
	medium	1254 (19.4)	29.8	(26.7; 33.1)		33.1	(29.7; 36.8)	
	high	2626 (40.7)	21.1	(18.8; 23.5)		20.4	(18.3; 22.6)	
Uganda (2016)	low	1576 (25.4)	34.8	(32.2; 37.5)	<0.001	40.3	(37.2; 43.3)	<0.001
	medium	1572 (25.3)	36.1	(33.0; 39.3)		36.5	(33.3; 39.8)	
	high	3063 (49.3)	26.1	(24.1; 28.2)		23.6	(21.7; 25.7)	
Zambia (2013)	low	2684 (35.7)	22.6	(20.5; 24.9)	<0.001	35.3	(32.9; 37.7)	<0.001
	medium	1254 (16.7)	18.5	(15.8; 21.5)		31.3	(28.2; 34.6)	
	high	3574 (47.6)	15.4	(13.8; 17.2)		21.5	(19.5; 23.7)	
Zimbabwe (2015)	low	588 (12.1)	30.6	(26.1; 35.6)	0.002	30.6	(25.8; 35.9)	<0.001
	medium	1133 (23.3)	27.8	(24.9; 31.0)		23.5	(20.6; 26.6)	
	high	3133 (64.5)	23.1	(21.0; 25.4)		18.0	(16.3; 19.9)	
**Middle East & North Africa**								
Egypt (2014)	low	944 (15.0)	19.7	(16.7; 23.1)	<0.001	23.3	(20.3; 26.6)	<0.001
	medium	1224 (19.5)	15.5	(13.3; 18.1)		17.9	(15.4; 20.6)	
	high	4122 (65.5)	10.1	(9.0; 11.3)		10.2	(9.1; 11.4)	
**Europe & Central Asia**								
Armenia (2015)	low	188 (5.8)	8.2	(4.5; 14.3)	<0.001	4.5	(2.1; 9.4)	<0.001
	medium	361 (11.2)	15.4	(11.0; 21.0)		10.2	(6.7; 15.3)	
	high	2683 (83.0)	4.5	(3.6; 5.6)		1.6	(1.1; 2.3)	
Kyrgyzstan (2012)	low	791 (18.3)	11.0	(8.1; 14.6)	<0.001	22.2	(18.5; 26.5)	<0.001
	medium	1272 (29.4)	14.6	(11.7; 18.0)		23.8	(20.5; 27.5)	
	high	2261 (52.3)	6.4	(5.1; 7.9)		11.9	(10.1; 14.0)	
**South Asia**								
Afghanistan (2015)	low	10852 (53.4)	35.9	(33.7; 38.1)	<0.001	51.3	(48.7; 53.9)	<0.001
	medium	6541 (32.2)	36.1	(33.1; 39.3)		45.7	(43.1; 48.2)	
	high	2923 (14.4)	25.7	(20.6; 31.6)		27.9	(24.2; 32.0)	
India (2015)	low	13470 (21.5)	17.0	(15.8; 18.2)	<0.001	33.5	(32.0; 35.0)	<0.001
	medium	13446 (21.5)	13.3	(12.4; 14.4)		29.8	(28.5; 31.1)	
	high	35638 (57.0)	7.7	(7.2; 8.2)		16.6	(15.9; 17.4)	

Nepal (2016)	low	226 (6.1)	7.2	(4.3; 11.6)	0.961	14.7	(10.5; 20.1)	0.036
	medium	807 (21.8)	7.7	(5.9; 10.1)		13.9	(11.3; 17.0)	
	high	2667 (72.1)	7.8	(6.4; 9.4)		10.2	(8.8; 11.8)	
Pakistan (2017)	low	1071 (33.5)	30.4	(25.6; 35.6)	<0.001	23.1	(19.5; 27.2)	<0.001
	medium	504 (15.7)	22.9	(17.6; 29.2)		17.7	(13.3; 23.1)	
	high	1626 (50.8)	16.2	(14.0; 18.7)		10.1	(8.4; 12.2)	
**East Asia & the Pacific**								
Cambodia (2014)	low	904 (28.2)	19.5	(16.1; 23.4)	0.0112	13.2	(10.6; 16.3)	0.057
	medium	873 (27.3)	20.6	(16.9; 24.9)		11.6	(8.9; 14.9)	
	high	1426 (44.5)	14.5	(11.9; 17.4)		8.9	(6.7; 11.6)	
Myanmar (2015)	low	494 (16.1)	9.8	(7.0; 13.7)	0.467	12.1	(9.0; 16.1)	0.010
	medium	1180 (38.4)	10.1	(8.0; 12.7)		12.7	(10.3; 15.7)	
	high	1400 (45.5)	8.4	(6.7; 10.5)		8.4	(6.7; 10.5)	
Philippines (2017)	low	226 (1.8)	30.2	(20.7; 41.8)	<0.001	14.9	(8.4; 24.9)	<0.001
	medium	1377 (11.0)	23.0	(19.8; 26.6)		9.3	(7.3; 11.7)	
	high	10904 (87.2)	10.8	(10.0; 11.8)		4.8	(4.2; 5.5)	
Timor Leste (2016)	low	2292 (64.2)	9.4	(7.7; 11.5)	<0.001	39.4	(36.6; 42.3)	<0.001
	medium	604 (16.9)	10.7	(7.6; 14.8)		34.6	(29.6; 40.0)	
	high	673 (18.9)	4.0	(2.6; 6.1)		18.8	(14.9; 23.5)	
**Latin America & Caribbean**								
Dominican Republic (2013)	low	17 (0.4)	57.6	(31.8; 79.8)	<0.001	56.2	(30.2; 79.2)	<0.001
	medium	92 (2.2)	43.0	(28.6; 58.9)		12.7	(7.4; 21.0)	
	high	3980 (97.3)	23.8	(21.7; 25.9)		13.2	(11.7; 14.9)	
Guatemala (2014)	low	165 (2.9)	12.5	(8.0; 18.9)	0.002	11.1	(6.9; 17.6)	0.002
	medium	499 (8.7)	21.1	(17.0; 25.9)		12.9	(9.9; 16.5)	
	high	5042 (88.4)	14.4	(13.1; 15.7)		8.0	(7.0; 9.1)	
Haiti (2016)	low	174 (4.6)	21.1	(15.1; 28.7)	<0.001	22.1	(15.3; 30.7)	0.076
	medium	438 (11.5)	28.9	(23.0; 35.7)		15.8	(11.9; 20.7)	
	high	3188 (83.9)	17.1	(15.3; 19.1)		14.0	(12.3; 15.9)	
Honduras (2011)	low	554 (5.3)	24.2	(20.1; 28.9)	<0.001	16.8	(13.4; 21.0)	<0.001
	medium	1124 (10.8)	28.0	(24.5; 31.8)		15.9	(19.1; 13.2)	
	high	8710 (83.8)	20.9	(19.8; 22.1)		10.4	(9.5; 11.3)	
Peru (2016)	low	114 (0.6)	12.1	(5.9; 23.4)	0.259	13.2	(6.9; 23.8)	0.045
	medium	343 (1.9)	14.2	(8.6; 22.6)		16.5	(10.8; 24.3)	
	high	17976 (97.5)	10.0	(9.2; 10.8)		10.4	(9.7; 11.1)	

***** p value based on χ^2 test for differences between groups

S5 Table. IPV prevalence by area of residence in each country.

Country	Area of residence	N (%)	Psychological IPV			Physical and/or Sexual IPV		
			%	(95% CI)	p value*	%	(95% CI)	p value*
**West & Central Africa**								
Burkina Faso (2010)	Urban	2694 (26.9)	10.6	(8.5; 13.2)	<0.001	11.9	(10.3;13.7)	<0.001
	Rural	7315 (73.1)	6.2	(5.5; 7.0)		8.5	(7.6; 9.5)	
Cameroon (2011)	Urban	1846 (46.1)	29.2	(26.7; 31.8)	0.002	29.3	(26.8; 31.9)	0.024
	Rural	2160 (53.9)	35.1	(32.2; 38.2)		33.6	(30.9; 6.4)	
Chad (2014)	Urban	834 (21.9)	16.8	(13.1; 21.3)	0.764	18.3	(14.5; 22.8)	0.617
	Rural	2980 (78.1)	16.1	(14.3; 18.2)		17.2	(15.1; 19.5)	
Congo DR (2013)	Urban	1622 (28.5)	25.5	(22.0; 29.4)	0.003	34.7	(30.3; 39.3)	0.134

	Rural	4069 (68.8)	31.4	(28.0; 35.0)		37.7	(34.1; 41.5)	
Cote d'Ivoire (2011)	Urban	1843 (36.7)	19.1	(16.2; 22.4)	<0.001	26.9	(24.1; 30.0)	<0.001
	Rural	3175 (63.3)	14.0	(12.3; 15.8)		20.2	(17.9; 22.7)	
Gabon (2012)	Urban	2588 (62.4)	26.3	(23.1; 29.9)	0.382	30.5	(27.1; 34.1)	0.013
	Rural	1559 (37.6)	28.2	(25.3; 31.3)		36.3	(33.2; 39.5)	
Gambia (2013)	Urban	1502 (42.4)	9.3	(7.0; 12.2)	0.267	6.9	(5.1; 9.4)	0.517
	Rural	2040 (57.6)	7.8	(6.0; 10.0)		7.7	(6.0; 9.8)	
Mali (2012)	Urban	869 (27.9)	24.3	(20.2; 28.9)	0.258	22.2	(18.2; 26.8)	0.001
	Rural	2251 (72.1)	26.7	(23.8; 29.7)		27.7	(24.7; 31.0)	
Nigeria (2013)	Urban	8139 (36.5)	16.4	(14.8; 18.1)	0.008	11.0	(9.8; 12.2)	0.918
	Rural	14166 (63.5)	14.7	(13.5; 15.9)		10.9	(10.0; 11.9)	
Senegal (2017)	Urban	1032 (38.8)	10.9	(8.2; 14.5)	0.140	13.7	(10.5; 17.9)	0.169
	Rural	1628 (61.2)	8.3	(6.8; 10.2)		11.1	(9.1; 13.4)	
Sierra Leone (2013)	Urban	1395 (32.3)	26.9	(22.4; 31.8)	<0.001	32.2	(27.4; 37.4)	0.021
	Rural	2920 (67.7)	18.2	(16.3; 20.2)		27.0	(24.7; 29.4)	
Togo (2013)	Urban	1848 (34.4)	18.8	(16.3; 21.6)	<0.001	11.7	(10.1; 13.6)	0.126
	Rural	3528 (65.6)	27.7	(25.3; 30.2)		13.4	(11.9; 15.0)	
**Eastern & Southern Africa**								
Angola (2015)	Urban	4343 (56.6)	26.0	(22.9; 29.3)	<0.001	26.7	(24.2; 29.3)	0.072
	Rural	3326 (43.4)	20.5	(18.0; 23.2)		24.2	(21.3; 27.2)	
Burundi (2016)	Urban	1215 (16.5)	12.9	(10.1; 16.3)	0.010	15.1	(12.0; 18.9)	<0.001
	Rural	6151 (83.5)	16.9	(15.7; 18.2)		29.4	(27.9; 30.9)	
Comoros (2012)	Urban	1038 (41.0)	6.0	(4.5; 7.8)	0.788	6.3	(4.8; 8.2)	0.056
	Rural	1491 (59.0)	6.3	(4.7; 8.3)		4.1	(2.8; 5.9)	
Ethiopia (2016)	Urban	1211 (25.7)	14.8	(10.8; 20.0)	0.005	11.9	(8.9; 15.8)	<0.001
	Rural	3509 (74.3)	21.4	(19.0; 24.0)		21.5	(19.3; 23.8)	
Kenya (2014)	Urban	1644 (36.4)	25.0	(21.7; 28.7)	0.283	25.0	(21.6; 28.8)	0.754
	Rural	2875 (63.6)	23.0	(21.0; 25.1)		25.7	(23.5; 27.9)	
Malawi (2015)	Urban	995 (18.4)	20.8	(16.7; 25.5)	0.240	21.1	(16.0; 27.3)	0.172
	Rural	4411 (81.6)	23.5	(21.8; 25.3)		24.7	(23.2; 26.4)	
Mozambique (2011)	Urban	1978 (34.0)	33.4	(30.7; 36.1)	0.001	31.2	(28.7; 33.8)	0.002
	Rural	3846 (66.0)	28.0	(25.9; 30.2)		26.1	(24.0; 28.3)	
Namibia (2013)	Urban	706 (53.0)	20.1	(16.5; 24.3)	0.431	19.3	(15.7; 23.4)	0.389
	Rural	627 (47.0)	22.3	(18.5; 26.5)		21.5	(17.4; 26.3)	
Rwanda (2014)	Urban	396 (20.8)	14.0	(10.5; 18.6)	0.025	18.2	(13.0; 24.8)	0.393
	Rural	1512 (79.2)	19.5	(17.5; 21.7)		21.1	(18.8; 23.5)	
South Africa (2016)	Urban	1410 (59.9)	10.1	(8.3; 12.3)	0.098	9.7	(7.7; 12.2)	0.157
	Rural	944 (40.1)	13.0	(10.6; 15.8)		12.4	(8.9; 16.9)	
Tanzania (2015)	Urban	2037 (26.8)	25.9	(23.3; 28.8)	0.034	27.3	(24.4; 30.4)	0.040
	Rural	5560 (73.2)	29.2	(27.2; 31.4)		30.5	(28.7; 32.4)	
Uganda (2016)	Urban	1555 (20.6)	24.4	(21.6; 27.4)	<0.001	22.2	(19.8; 24.8)	<0.001
	Rural	5981 (79.4)	30.8	(29.1; 32.5)		31.9	(30.1; 33.8)	
Zambia (2013)	Urban	3920 (41.6)	17.5	(15.5; 19.6)	0.655	25.5	(23.2; 27.9)	0.154
	Rural	5496 (58.4)	17.9	(16.6; 19.4)		27.3	(25.7; 29.0)	
Zimbabwe (2015)	Urban	2419 (41.7)	23.6	(20.8; 26.6)	0.957	19.8	(17.8; 21.9)	0.937
	Rural	3381 (58.3)	23.5	(21.8; 25.3)		19.9	(18.0; 21.9)	
**Middle East & North Africa**								
Egypt (2014)	Urban	2989 (44.7)	13.3	(11.8; 15.0)	0.712	12.6	(11.1; 14.3)	0.039
	Rural	3704 (55.3)	12.9	(11.7; 14.3)		14.8	(13.5; 16.2)	
Jordan (2017)	Urban	5452 (79.6)	16.1	(14.5; 17.9)	0.880	14.0	(12.3; 15.9)	0.164
	Rural	1400 (20.4)	16.3	(13.3; 19.9)		11.9	(9.3; 15.2)	
**Europe & Central Asia**								

Armenia (2015)	Urban	2055 (58.1)	5.6	(4.3; 7.3)	0.056	3.1	(2.2; 4.4)	0.178
	Rural	1485 (41.9)	7.6	(6.3; 9.1)		4.1	(3.2; 5.4)	
Kyrgyzstan (2012)	Urban	1491 (30.9)	8.7	(6.9; 10.9)	0.046	15.5	(13.1; 18.3)	0.117
	Rural	3341 (69.1)	11.3	(9.9; 13.0)		18.0	(15.9; 20.3)	
Tajikistan (2017)	Urban	2162 (40.7)	11.6	(9.6; 13.9)	0.043	16.2	(13.9; 18.7)	0.005
	Rural	3151 (59.3)	13.8	(12.0; 15.9)		19.9	(17.4; 22.7)	
**South Asia**								
Afghanistan (2015)	Urban	5306 (24.9)	33.8	(30.5; 37.4)	0.626	37.4	(33.3; 41.7)	<0.001
	Rural	16018 (75.1)	34.6	(32.7; 36.6)		48.5	(46.1; 51.0)	
India (2015)	Urban	19469 (29.5)	10.1	(9.3; 11.1)	<0.001	19.4	(18.0; 20.9)	<0.001
	Rural	46544 (70.5)	12.1	(11.6; 12.7)		25.9	(25.3; 26.7)	
Maldives (2016)	Urban	349 (10.3)	8.0	(4.9; 12.8)	0.730	5.4	(3.2; 9.0)	0.913
	Rural	3039 (89.7)	7.4	(6.2; 8.8)		5.6	(4.6; 6.8)	
Nepal (2016)	Urban	2380 (62.2)	7.4	(6.0; 9.2)	0.601	10.2	(8.7; 12.0)	0.055
	Rural	1446 (37.8)	8.1	(6.4; 10.1)		12.8	(10.7; 15.1)	
Pakistan (2017)	Urban	1646 (49.8)	16.9	(14.2; 19.9)	0.003	11.0	(9.0; 13.3)	0.001
	Rural	1657 (50.2)	22.9	(19.8; 26.2)		16.6	(14.3; 19.3)	
**East Asia & the Pacific**								
Cambodia (2014)	Urban	887 (25.4)	12.3	(9.0; 16.6)	0.009	5.5	(3.7; 8.0)	<0.001
	Rural	2612 (74.6)	18.2	(16.2; 20.3)		11.9	(10.3; 13.8)	
Myanmar (2015)	Urban	832 (24.3)	10.7	(8.0; 14.2)	0.689	8.9	(6.4; 12.2)	0.094
	Rural	2593 (75.7)	10.1	(8.5; 11.8)		11.8	(10.2; 13.5)	
Philippines (2017)	Urban	4336 (32.8)	11.7	(10.3; 13.3)	0.016	5.0	(4.2; 6.1)	0.241
	Rural	8879 (67.2)	13.9	(12.4; 15.5)		5.8	(5.0; 6.6)	
Timor Leste (2016)	Urban	1025 (27.7)	10.9	(7.7; 15.3)	0.100	26.7	(22.7; 31.1)	<0.001
	Rural	2669 (72.3)	8.1	(6.9; 9.6)		37.5	(34.8; 40.2)	
**Latin America & Caribbean**								
Colombia (2015)	Urban	18138 (73.0)	30.1	(28.9; 31.3)	0.854	33.9	(32.7; 35.2)	0.002
	Rural	6724 (27.0)	29.9	(28.3; 31.6)		30.9	(29.2; 32.6)	
Dominican Republic (2013)	Urban	4097 (70.6)	27.1	(25.0; 29.4)	<0.001	16.3	(14.6; 18.1)	0.114
	Rural	1706 (29.4	21.2	(17.6; 25.4)		13.7	(11.3; 16.4)	
Guatemala (2014)	Urban	2708 (41.6)	16.7	(14.9; 18.7)	<0.001	8.2	(6.8; 9.7)	0.527
	Rural	3804 (58.4)	12.7	(11.5; 14.2)		8.7	(7.5; 10.1)	
Haiti (2016)	Urban	1469 (34.0)	19.7	(17.1; 22.5)	0.052	15.7	(13.1; 18.7)	0.027
	Rural	2853 (66.0)	16.5	(14.5; 18.7)		12.4	(10.7; 14.3)	
Honduras (2011)	Urban	4810 (38.5)	22.6	(21.0; 24.2)	<0.001	11.5	(10.4; 12.6)	0.109
	Rural	7684 (61.5)	18.6	(17.5; 19.7)		10.3	(9.4; 11.3)	
Peru (2016)	Urban	14460 (68.5)	11.0	(10.1; 11.9)	0.010	11.1	(10.2; 11.9)	0.081
	Rural	6655 (31.59)	9.2	(8.3; 10.2)		10.0	(9.1; 11.0)	

***** p value based on χ^2 test for differences between groups
